# Transcriptomic alterations underlying metaplasia into specific metaplastic components in metaplastic breast carcinoma

**DOI:** 10.1186/s13058-023-01608-5

**Published:** 2023-01-27

**Authors:** Huang-Chun Lien, Chia-Lang Hsu, Yen-Shen Lu, Tom Wei-Wu Chen, I.-Chun Chen, Yu-Chia Li, Chiun-Sheng Huang, Ann-Lii Cheng, Ching-Hung Lin

**Affiliations:** 1 Department of Pathology, National Taiwan University Hospital, Taipei, Taiwan; 2 Graduate Institute of Pathology, National Taiwan University, Taipei, Taiwan; 3 Graduate Institute of Oncology, National Taiwan University, Taipei, Taiwan; 4 Graduate Institute of Medical Genomics and Proteomics, National Taiwan University, Taipei, Taiwan; 5 Department of Medical Research, National Taiwan University Hospital, Taipei, Taiwan; 6 Department of Oncology, National Taiwan University Hospital, Taipei, Taiwan; 7 Department of Internal Medicine, National Taiwan University, Taipei, Taiwan; 8 Department of Medical Oncology, Cancer Center Branch, National Taiwan University Hospital, Taipei, Taiwan; 9 Department of Surgery, National Taiwan University Hospital, Taipei, Taiwan

**Keywords:** Metaplastic breast carcinoma, Invasive carcinoma of no special type, Spindle carcinomatous component, Matrix-producing component, Rhabdoid component, Squamous carcinomatous component, Gene expression profile

## Abstract

**Background:**

Metaplastic breast carcinoma (MpBC) typically consists of carcinoma of no special type (NST) with various metaplastic components. Although previous transcriptomic and proteomic studies have reported subtype-related heterogeneity, the intracase transcriptomic alterations between metaplastic components and paired NST components, which are critical for understanding the pathogenesis underlying the metaplastic processes, remain unclear.

**Methods:**

Fifty-nine NST components and paired metaplastic components (spindle carcinomatous [SPS], matrix-producing, rhabdoid [RHA], and squamous carcinomatous [SQC] components) were microdissected from specimens obtained from 27 patients with MpBC for gene expression profiling using the NanoString Breast Cancer 360 Panel on a NanoString nCounter FLEX platform. BC360-defined signatures were scored using nSolver software.

**Results:**

Hierarchical clustering and principal component analysis revealed a heterogeneous gene expression profile (GEP) corresponding to the NST components, but the GEP of metaplastic components exhibited subtype dependence. Compared with the paired NST components, the SPS components demonstrated the upregulation of genes related to stem cells and epithelial–mesenchymal transition and displayed enrichment in claudin-low and macrophage signatures. Despite certain overlaps in the enriched functions and signatures between the RHA and SPS components, the specific differentially expressed genes differed. We observed the RHA-specific upregulation of genes associated with vascular endothelial growth factor signaling. The chondroid matrix-producing components demonstrated the upregulation of hypoxia-related genes and the downregulation of the immune-related MHC2 signature and the TIGIT gene. In the SQC components, *TGF-β* and genes associated with cell adhesion were upregulated. The differentially expressed genes among metaplastic components in the 22 MpBC cases with one or predominantly one metaplastic component clustered paired NST samples into clusters with correlation with their associated metaplastic types. These genes could be used to separate the 31 metaplastic components according to respective metaplastic types with an accuracy of 74.2%, suggesting that intrinsic signatures of NST may determine paired metaplastic type. Finally, the EMT activity and stem cell traits in the NST components were correlated with specimens displaying lymph node metastasis.

**Conclusions:**

We presented the distinct transcriptomic alterations underlying metaplasia into specific metaplastic components in MpBCs, which contributes to the understanding of the pathogenesis underlying morphologically distinct metaplasia in MpBCs.

**Supplementary Information:**

The online version contains supplementary material available at 10.1186/s13058-023-01608-5.

## Introduction

Metaplastic breast carcinoma (MpBC) is a rare cancer that accounts for less than 1% of primary breast malignancies [1]. In general, MpBC is biphasic and comprises both carcinomatous and sarcomatous components. The carcinomatous component is typically carcinoma of no special type (NST) in which squamous metaplasia may occur to a variable extent. The sarcomatous components can exhibit spindled, rhabdomyoid and matrix-producing histomorphologies, among others [2]. Occasionally, the sarcomatous component predominantly consists of noncohesive cells with large eccentric nuclei, prominent nucleoli, and intracytoplasmic inclusion-like features but lacks myogenic markers expression, reminiscent of rhabdoid metaplasia. Although the majority of MpBCs do not express estrogen receptors (ERs), progesterone receptors (PRs), or human epidermal growth factor receptor 2 (HER2), they are typically more aggressive and less responsive to chemotherapy than conventional triple-negative breast cancers (TNBCs) [1, 3, 4]. The multivariate analysis of a prior study demonstrated that the prognosis of MpBCs was dependent on the metaplastic subtype, with spindle cell carcinoma demonstrating particularly aggressive behavior [5]. This presents a clinical challenge that highlights the need to investigate the pathogenesis underlying the distinct metaplastic components of MpBCs.

The histopathology and underlying pathogenesis of MpBC, for which a single case may contain multiple carcinomatous and sarcomatous components, has long been a topic of scholarly interest. A growing body of evidence has indicated that MpBCs share a genetic background with in situ and invasive carcinoma and metaplastic sarcomatous components, with these sarcomatous components being derived from NST through various metaplastic processes [6–10]. Despite the lack of a genetic basis underlying these histologic subtypes [10, 11], studies have revealed distinct transcriptomic and proteomic profiles to be correlated with different MpBC subtypes [11–13]. However, intercase heterogeneity may complicate inferences of the pathogenesis underlying distinct metaplastic changes. Because these sarcomatous components are metaplastically transformed from NST, a direct comparison between NST and paired metaplastic components, which is critical for elucidating the pathogenesis underlying distinct metaplastic changes, has not yet been made. Herein, we analyzed 59 dissectible NST components and paired metaplastic components, including spindle carcinomatous (SPS), matrix-producing, rhabdoid (RHA), and squamous carcinomatous (SQC) components, collected from 27 patients with MpBC. We used hybridization-based transcriptomic analysis technology to identify the gene expression profile (GEP) underlying the metaplasia of NST into distinct metaplastic components.

## Materials and methods

### Tumor samples and needle-assisted microdissection

The study protocol was approved by the Institutional Review Board of National Taiwan University Hospital (Approval No. 201711051RINC). From the Department of Pathology of the hospital, we retrieved formalin-fixed, paraffin-embedded (FFPE) surgical specimens collected between 1998 and 2019 from 27 patients with biphasic MpBC who had dissectible tumor components. The 27 cases comprised metaplastic carcinoma with heterologous mesenchymal differentiation (*n* = 12), spindle cell carcinoma (*n* = 9), squamous cell carcinoma (*n* = 3), and mixed metaplastic carcinoma (*n* = 3). Ten 10-µm hematoxylin-counterstained slides of each dissectible NST component and paired metaplastic component were prepared for needle-assisted microdissection, in which a 27-gauge needle was used under 40 × magnification. A total of 59 dissected tumor components were collected for RNA extraction: these comprised NST components (*n* = 27) and paired metaplastic components, namely SPS (*n* = 12), RHA (*n* = 6), matrix-producing (chondroid, *n* = 9; osteoid, *n* = 1), and SQC (*n* = 4). All the six RHA components showed no convincing staining for myogenic markers (desmin and myogenin). The chondroid and osteoid matrix-producing components are hereafter referred to as MAT and OGS, respectively. The tumor size and the status of lymph node metastasis were recorded for all specimens. Lymph node metastasis was observed in 10 cases of MpBC with both carcinomatous and sarcomatous components. Seven of these cases involved only carcinomatous components in the metastatic lymph nodes. The other three cases involved both carcinomatous and sarcomatous components, with the carcinomatous components being predominant.

### Immunohistochemistry

ER (SP1, Ventana, Tucson, AZ, USA), PR (1E2, Ventana), and HER2 (4B5, Ventana) staining was performed using the Ventana iVIEW DAB detection kit with an autoimmunostainer (Ventana BenchMark). Specimens demonstrating HER2 (2 +) were further tested for HER2 through fluorescence in situ hybridization (FISH; PathVysion, Abbott, Abbott Park, IL, USA). Immunohistochemistry was performed to verify the presence of differentially expressed genes in the metaplastic components, the intrinsic gene sets of NST components, and the differentially expressed genes associated with lymph node metastasis. Primary antibodies against EPAS1 (SC13596; Santa Cruz Biotech, Dallas, TX, USA), SLC2A1 (SC377228), IL1RA (SC374084), CAV1 (EP353; Bio SB, Santa Barbara, CA, USA), FBN1 (HPA021057; Sigma-Aldrich, St. Louis, MO, USA), HAPLN1 (HPA019482), COL9A3 (HPA040125), PYCARD (HPA054496), PDGFRA (HPA004947), NCAM1 (MRQ-42, Ventana), and SOX10 (SP267, Cell Marque, Rocklin, CA, USA) were used.

### Tumor RNA isolation and gene expression assay

RNA isolation was conducted using the Roche High Pure FFPE RNA Isolation Kit (Roche Molecular Systems, Pleasanton, CA, USA). To ensure sample purity (optical density 260/280 nm; ratio 1.7–2.5), the RNA concentration was estimated using the NanoDrop ND-1000 spectrophotometer and the Qubit 3.0 Fluorometer (Thermo Fisher Scientific, Waltham, MA, USA). The GEP was analyzed using the NanoString Breast Cancer 360 (BC360) Panel on a NanoString nCounter FLEX platform (NanoString Technologies, Seattle, WA, USA). The BC360 Panel contains 770 genes across 23 breast cancer-related pathways and processes as well as 30 signatures for measuring tumor and immune activities [14, 15]. Intrinsic molecular subtypes of PAM50 were used to classify breast cancer into four subtypes (luminal A, luminal B, HER2-enriched, and basal-like) [16, 17]. Risk of recurrence (ROR) scores are derived from the expression profile of 46 PAM50 genes with a weighted sum of the proliferation score, the four subtype correlations and tumor size information to calculate a score between 0 and 100 [18, 19]. The whole tumor size was used in this calculation, so the comparison of ROR score between NST and its paired metaplastic component mainly indirectly compared the expression of proliferation-associated genes of these two components. BC360-defined signatures were scored using nSolver software (NanoString Technologies).

### Gene set enrichment analysis

Gene set enrichment analysis (GSEA) was performed using the enrichment analysis function in the clusterProfiler R package. The gene sets used in the GSEA were obtained from the c2.cp, c5.bp, and hallmark collections in the Molecular Signatures Database (MSigDB; version 7.0). We used a preranked GSEA to analyze gene lists ranked by the − log_10_(*p*) * sign(log_2_ fold-change), where *p* was derived from paired *t* tests for paired samples or from *t* tests for unpaired samples.

### Data availability

Raw data from this study were deposited in the Gene Expression Omnibus (GEO) with Accession Number GSE212245.

### Statistical analysis

Processing, analyses, and plotting were conducted using R3.5.2 software (http://www.r-project.org/). The paired *t* test, *t* test, and analysis of variance (ANOVA) were applied to paired samples, unpaired samples, and multigroup samples, respectively. A hierarchical clustering analysis was performed using the pheatmap R package with the clustering distance set to the “euclidean” default and with the clustering method set to “ward.D2.”

## Results

### Clinicopathological characteristics of the tumor samples

**Fig. 1:**
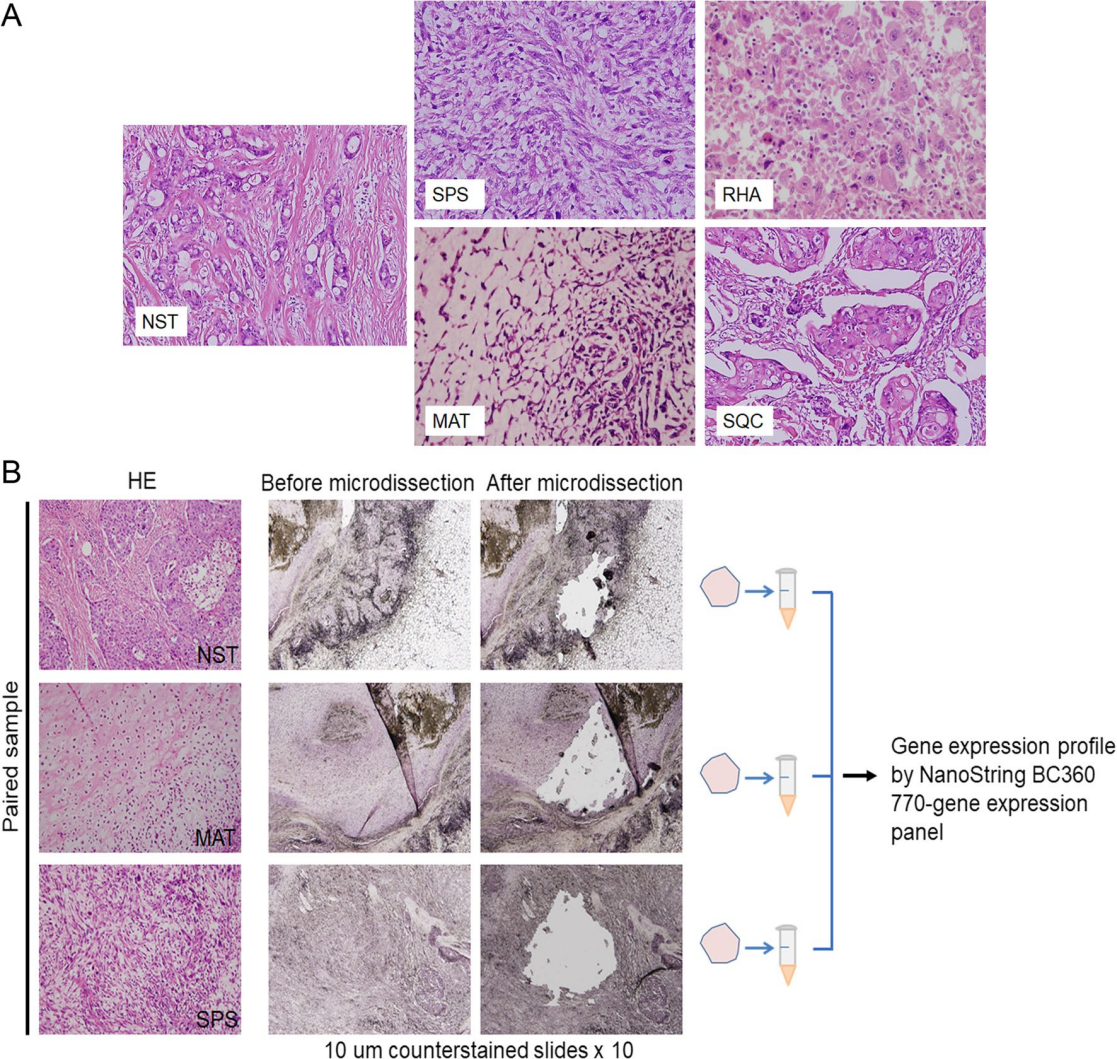
Distinct histomorphological patterns of MpBC tumors and the workflow of sample collection and gene expression analysis. **A** Representative histomorphology of NST and components conforming to four major metaplastic patterns, namely SPS, RHA, MAT, and SQC. **B** Workflow of tissue collection and gene expression analysis, conducted using the NanoString BC360 Panel (770 genes). H&E, hematoxylin and eosin

**Fig. 2:**
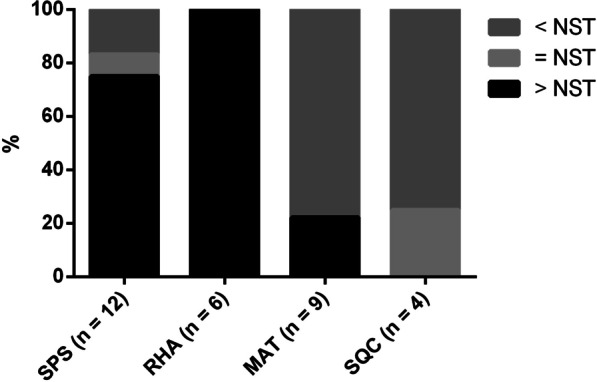
Comparison of PAM50-based ROR scores between metaplastic tumor components and paired NST components

In total, 59 dissected NST components and paired metaplastic components were collected and subjected to gene expression profiling by using the NanoString BC360 Panel (Fig. 1). The clinicopathological characteristics and molecular intrinsic subtypes of PAM50 are presented with ROR scores in Table 1, and the information of patients’ treatment and outcome is shown in Additional file 4: Table S1. All 31 metaplastic components were classified as the basal-like subtype and were determined to be ER− , PR− , and HER2− . Four and two NST components were classified as the HER2-enriched and luminal A subtypes, respectively. The components were consistent in immunohistochemistry, except case BT83, which was classified as the HER2-enriched subtype and HER2 2+ in immunohistochemistry testing, but HER2 testing by FISH was negative. The remaining 21 NST components were classified as the basal-like subtype, with 19 components being ER− , PR− , and HER2− and the other 2 components demonstrating 5% and 20% ER in immunohistochemical staining. Notably, compared with that of the paired NST components, the ROR score was higher or equal in 83.3% (10/12) of SPS components and in 100% (6/6) of RHA components. However, it was lower in 77.8% (7/9) of MAT components and was lower or equal in 75% (3/4) and 25% (1/4) of SQC components, respectively (Fig. 2).

**Table 1:** Clinicopathological characteristics, intrinsic molecular subtypes of PAM50, and PAM50-based ROR of the 59 NST and paired metaplastic components from 27 patients with MpBC

Case	MpBC subtype	Histologic components	Dissectible components	PAM50 subtype	ROR	Age	TNM stage	Stage	ER/PR/HER2	SBR grade
BT5	SPC	SPS/NST	SPS/NST	BL/BL	75/62	73	T3N0M0	IIB	(−/ −/ −)	(III, III)
BT34	SPC	SPS/NST	SPS/NST	BL/BL	80/80	63	T2N0M0	IIA	(−/ −/− in SPS; 5%/ −/− in NST)	(III, III)
BT42	SPC	SPS/SQC/NST	SPS/NST	BL/BL	52/50	53	T2N0M0	IIA	(−/ −/ −)	(III, III)
BT46	SPC	SPS/NST	SPS/NST	BL/BL	55/62	39	T1N0M0	IA	(−/ −/ −)	(III, III)
BT61	SPC	SPS/NST	SPS/ NST	BL/HER2e	64/ 85	69	T2N0M0	IIA	(−/ −/− in SPS; −/ −/ + in NST)	(III, III)
BT65	SPC	SPS/NST	SPS/NST	BL/BL	72/ 56	46	T1N0M0	IA	(−/ −/ −)	(III, III)
BT91	SPC	SPS/SQC/NST	SPS/NST	BL/BL	74/62	91	T4N1M0	IIIB	(−/ −/− in SPS; 20%/ −/− in NST)	(III, III)
BT95	MMC	SPS/MAT/NST	SPS/MAT/NST	BL/BL/BL	76/68/63	46	T3N1M0	IIIA	(−/ −/ −)	(III, II, III)
BT15	MMC	SPS/ RHA/ MAT/ NST	SPS/RHA/NST	BL/BL/BL	62/70/60	47	T3N0M0	IIB	(−/ −/ −)	(III, III, III)
BT118	MMC	SPS/OGS/MAT/ NST	SPS/OGS/MAT/NST	BL/BL/BL/BL	77/67/49/62	74	T2N0M0	IIA	(−/ −/ −)	(III, III, III, III)
BT122	SPC	SPS/NST	SPS/NST	BL/BL	70/59	54	T3N1M0	IIIA	(−/ −/ −)	(III, III)
BT127	SPC	SPS/SQC/NST	SPS/SQC/NST	BL/BL/ BL	86/56/71	60	T2N1M0	IIB	(−/ −/ −)	(III, III, III)
BT3	SCC	SQC/NST	SQC/NST	BL/LA	51/51	72	T4N3M0	IIIC	(−/ −/− in SCC; 70%/ −/− in NST)	(III, II)
BT83	SCC	SQC/NST	SQC/NST	BL/HER2e	56/70	48	T4N3M0	IIIC	(−/ −/− in SCC; 60%/ −/ −* in NST)	(III, II)
BT131	SCC	SQC/NST	SQC/ NST	BL/HER2e	56/90	49	T2N0M0	IIA	−/ −/− in SCC; −/ −/ + in NST	(III, III)
BT23	MHM	RHA/NST	RHA/NST	BL/BL	87/59	51	T3N0M0	IIB	−/ −/ −	(III, III)
BT57	MHM	RHA/NST	RHA/NST	BL/BL	62/54	72	T2N1M0	IIB	−/ −/ −	(III, III)
BT69	MHM	RHA/NST	RHA/NST	BL/HER2e	76/68	81	T3N1M0	IIIA	−/ −/− in RHA; −/ −/ + in NST	(III, III)
BT137	MHM	RHA/NST	RHA/NST	BL/LA	79/39	48	T1N0M0	IA	−/ −/− in RHA; 10%/ 5%/− in NST	(III, III)
BT79	MHM	RHA/SQC/NST	RHA/NST	BL/BL	79/67	44	T3N1M0	IIIA	−/ −/ −	(III, III)
BT6	MHM	MAT/NST	MAT/NST	BL/BL	58/77	43	T3N2M0	IIIB	−/ −/ −	(II, III)
BT44	MHM	MAT/SQC/NST	MAT/NST	BL/BL	45/62	56	T3N0M0	IIB	−/ −/ −	(II, II)
BT64	MHM	MAT/NST	MAT/NST	BL/BL	50/58	60	T2N0M0	IIA	−/ −/ −	(III, III)
BT71	MHM	MAT/NST	MAT/NST	BL/BL	50/57	54	T2N0M0	IIA	−/ −/ −	(III, III)
BT85	MHM	MAT/NST	MAT/NST	BL/BL	44/65	72	T1N0M0	IA	−/ −/ −	(II, III)
BT100	MHM	MAT/NST	MAT/NST	BL/BL	69/75	40	T3N1M0	IIIA	−/ −/ −	(III, III)
BT130	MHM	MAT/NST	MAT/NST	BL/BL	59/33	43	T3N1M0	IIIA	−/ −/ −	(II, III)

*IHC2+, HER2 FISH−

*BL* basal-like, *HER2e* HER2-enriched, *LA* luminal A, *MHM* metaplastic carcinoma with heterologous mesenchymal differentiation, *MMC* mixed metaplastic carcinoma, *ROR* risk of recurrence, *SBR* Scarff-Bloom-Richardson, *SCC* squamous cell carcinoma, *SPC* spindle cell carcinoma

### Gene expression of metaplastic components revealing subtype dependence

**Fig. 3:**
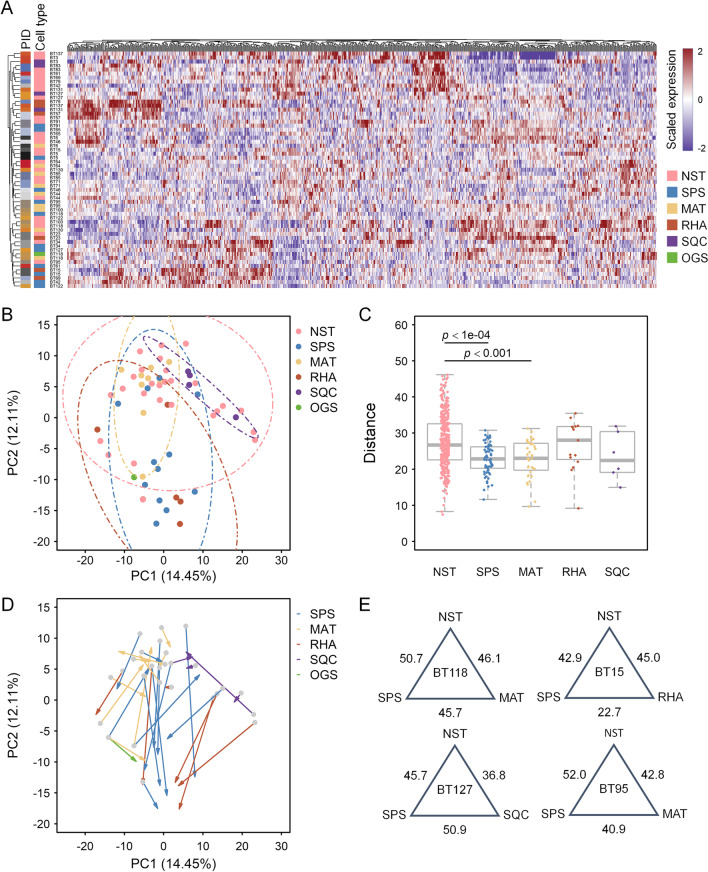
Transcriptomic differences among the NST components and metaplastic components. **A** Heat map of 758 genes (excluding housekeeping genes and probes for internal control) across 59 specimens obtained from 27 patients with MpBC. PID, patient identification. **B** PCA plot of unsupervised clustering among the 59 specimens. **C** Distribution of between-sample differences quantified based on the Euclidean distances of expression profiles in the NST components and distinct metaplastic components. **D** PCA plot identical to **B** but with NST components and paired metaplastic component linked by arrows, illustrating subtype-specific differentiation. **E** Euclidean distance based on expression profiles among NST components and metaplastic components in four patients with MpBC involving multiple metaplastic components

We performed hierarchical clustering and principal component analysis (PCA) to illustrate the relationship among the 59 components on the basis of the overall GEPs (Fig. 3A-E and Additional file 5: Table S2). Although hierarchical clustering revealed a modest distinction among the metaplastic component subtypes, intracase clustering of GEPs was noted in 14 of the 27 MpBC cases (Fig. 3A), suggesting that the overall intercase tumor heterogeneity was high. PCA indicated relatively clustered GEPs in each metaplastic subtype, but the NST specimens displayed greater heterogeneity than the specimens corresponding to the metaplastic subtypes (Fig. 3B). The high intercase heterogeneity among the NST components was further demonstrated by the relatively wide range of Euclidean distances between those components in the GEPs (Fig. 3C). The distribution of the specimens in the PCA further revealed a modest overlap between the RHA and SPS components, differentiating them from the MAT and SQC components (Fig. 3B). However, when metaplastic subtype-specific differentiation was considered, GEP differences between NST components and paired SPS or RHA components were greater than those between NST components and paired MAT or SQC components (Fig. 3D). This finding was supported by the fact that a larger Euclidean distance was observed in the GEPs between NST components and paired SPS or RHA components than between paired MAT or SQC components in the four MpBC cases with multiple metaplastic components (Fig. 3E). These results suggest that the GEPs of metaplastic components are subtype dependent.

### Metaplastic component-specific expressed genes as potential indicators of distinct intrinsic molecular characteristics

**Fig. 4:**
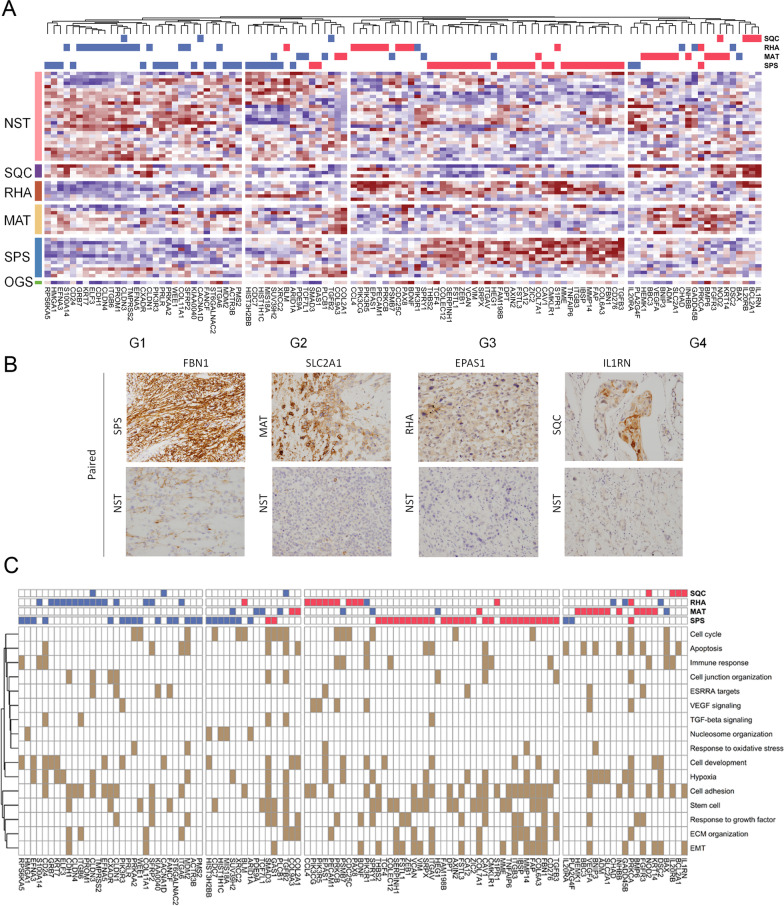
Differences in differentially expressed genes between the NST components and paired metaplastic components. **A** Heat map of differences in differentially expressed genes between these components (paired *t* test, *p* < 0.01) clustered into four groups. The top panel presents genes significantly downregulated (blue) or significantly upregulated (red) in corresponding cell types compared with the corresponding genes in the NST components. **B** Immunohistochemical validation of *FBN*, *SLC2A1*, *EPAS1*, and *IL1RN*, representative differentially expressed genes in the SPS, MAT, RHA, and SQC components, respectively. **C** Heat map showing gene–function associations. The order of the genes was identical to that in **A**. The top panel presents genes significantly downregulated (blue) or significantly upregulated (red) in corresponding cell types compared with the corresponding genes in the NST components

To investigate the functional difference between NST components and metaplastic components, the genes differentially expressed between the 59 NST components and paired metaplastic components were identified. Of these genes, 55 (31 upregulated, 24 downregulated) were identified in SPS components, 22 (14 upregulated, 8 downregulated) were identified in MAT components, 31 (12 upregulated, 19 downregulated) were identified in RHA components, and 7 (4 upregulated and 3 downregulated) were identified in SQC components (paired *t* test, *p* < 0.01). These differentially expressed genes were clustered into four groups (G1–G4) according to their expression patterns across all samples (Fig. 4A and Additional file 6: Table S3). To verify the genes that were differentially upregulated between the NST components and the paired SPS, MAT, RHA, or SQC components, we selected a representative gene from each of the four metaplastic components for immunohistochemical analysis. *FBN1*, *SLC2A1*, *EPAS1*, and *IL1RN* were differentially expressed in the SPS, MAT, RHA, and SQC components, respectively, and were subjected to immunohistochemical verification (Fig. 4B). The biological processes involving the differentially expressed genes are displayed in Fig. 4C. Most genes upregulated in the SPS components belonged to G3. These genes were enriched in functions such as stem cell, cell adhesion, epithelial–mesenchymal transition (EMT), extracellular matrix organization, and growth factor responses (Fig. 4C). Most SPS-specific downregulated genes, which belonged to G1 and G2, were associated with nucleosome organization (e.g., *HMGA1*, *HIST3H2BB*, *MIS18A*, and *ARID1A*) as well as with cell cycle (e.g., *PRKAA2*, *WEE1*, *MDM2*, *CDC7*, and *XRCC2*) and cell development (e.g., *EFNA3*, *CD24*, and *PIK3R3*). The upregulated and downregulated genes corresponding to the RHA components demonstrated an overall similarity to those of the SPS components, which belonged to G3 and G1, respectively. Furthermore, despite some overlap in enriched functions, such as cell adhesion, cell development, stem cell upregulation (e.g., RHA-specific gene *EPAS1* [20]), and EMT (e.g., RHA-specific gene *BDNF* [21]), the specific differentially expressed genes differed between the RHA and SPS components (Fig. 4A and C). By contrast, certain RHA-specific upregulated genes were associated with vascular endothelial growth factor (VEGF) signaling. Moreover, some RHA-specific downregulated genes were linked to cell adhesion and hypoxia. In addition, the SPS-specific downregulated genes that were associated with nucleosome organization and cell cycle were not downregulated in the RHA components. Mainly belonging to G4, the upregulated genes corresponding to the MAT components were associated with functions such as hypoxia (namely *VEGFA*, *BNIP3*, *ADM*, and *SLC2A1*) and apoptosis (namely *BBC3*, *BNIP3*, *INHBB*, *FGFR3*, and *COL2A1*) (Fig. 4A, C). The downregulated genes corresponding to the MAT components, primarily belonging to G2, were associated with cell-cycle control (e.g., *BAX*, *SPRY1*, *PSMB7*, and *PLCB1*). Mainly belonging to G4, the upregulated genes in the SQC components, including *NOD2*, *IL20RB*, *BCL2A1*, and *IL1RN*, were linked to apoptosis, immune responses, and cell adhesion. Overall, despite some overlap between SPS and RHA components, the functions of the differentially expressed genes in each metaplastic component revealed distinct intrinsic molecular characteristics.

### Differentially expressed signatures in specific metaplastic components

**Fig. 5:**
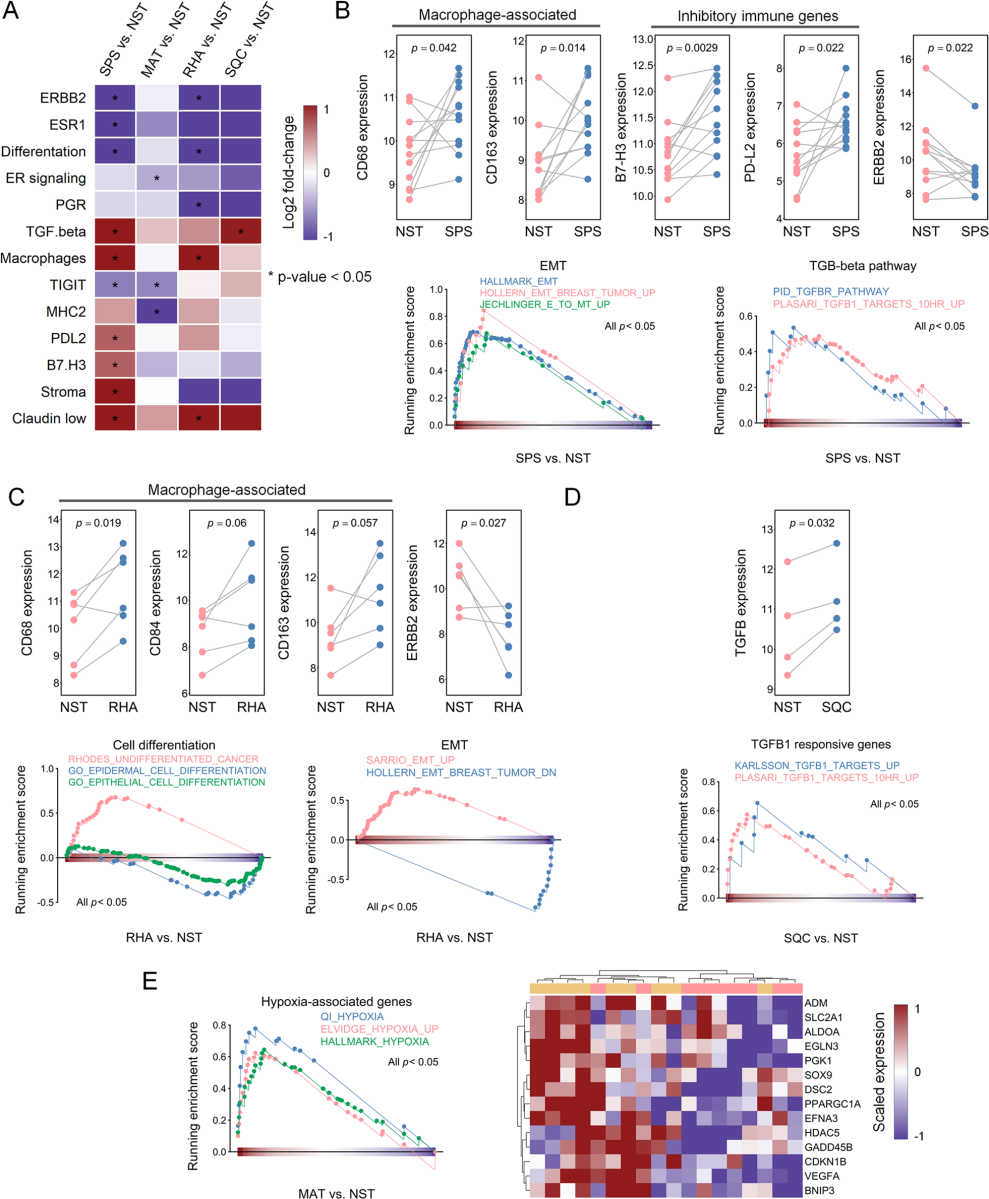
Differentially expressed BC360-defined signatures of the NST components and paired metaplastic components.** A** Heat map of differences in the expression of BC360-defined signatures in the NST and paired metaplastic components, with signatures exhibiting significant differences (paired *t* test, *p* < 0.05) labeled with asterisks. Log2 fold-change indicates log2 fold-change of the gene signature score of the metaplastic component with respect to the NST component. Plotted figures demonstrate the differences in the expression of representative genes and the GSEA-based activity of gene sets in the **B** SPS, **C** RHA, **D** SQC, and **E** MAT with respect to their paired NST components. A paired *t* test was conducted to evaluate differences in expression. Gene sets were obtained from MSigDB. **E** Heat map (right panel) of the expression of leading-edge genes from the GSEA results for the NST and MAT components (left panel)

To identify the gene expression signatures underlying metaplastic processes in MpBC, the differentially expressed signatures defined by the BC360 Panel were identified (paired *t* test, *p* < 0.05) and visualized as a heat map (Fig. 5A and Additional file 7: Table S4). Consistent with the findings presented in Fig. 4A, C, we observed the expression of some overlapping signatures in the SPS and RHA components. The expression of the claudin-low, stroma, and macrophage signatures and various genes (including *TGF-β* and inhibitory immune genes *PD-L2* and *B7.H3*) was higher in the SPS components than in the NST components (Fig. 5A, B). By contrast, differentiation signatures and genes including *ESR1*, *ERBB2*, and T cell immunoreceptor with Ig and immunoreceptor tyrosine-based inhibitory motif domains (*TIGIT*) were downregulated in the SPS components. We further performed a GSEA on external gene sets related to EMT, which in turn is related to claudin-low signatures and TGF-β signaling. The SPS components exhibited high activity of EMT and TGF-β signaling (Fig. 5B). Compared with the NST components, the RHA components had a more distinct claudin-low signature and greater macrophage abundance but a less distinct differentiation signature. This was further demonstrated at the gene expression level of macrophage-related genes (*CD84*, *CD163*, and *CD68*) and validated through GSEA on gene sets linked to the EMT and cell differentiation (Fig. 5C). *TGF-β* was upregulated in the SQC components; this finding was validated through the GSEA of TGF-β-responsive genes (Fig. 5A, D). As presented in Fig. 4A, because many MAT-specific genes were linked to hypoxia, we examined the expression of hypoxia-responsive genes collected from MSigDB. These genes were relatively highly expressed in the MAT components compared with in the NST components (Fig. 5E). Compared with those in the NST components, the MHC2 signature and *TIGIT* in the MAT components were downregulated (Fig. 5A). These results were consistently obtained for the four cases with multiple metaplastic components, with claudin-low, macrophage, and *TGF-β* signatures scores being higher and differentiation signature scores being lower in the SPS and RHA components than in the paired NST components. Two specimens contained MAT components, and both had higher hypoxia signature scores than the paired NST components. The only case with SQC component had a higher *TGF-β* score than did the paired NST component (Additional file 8: Table S5).

### Intrinsic gene expression of NST as a determinant of metaplastic type

**Fig. 6:**
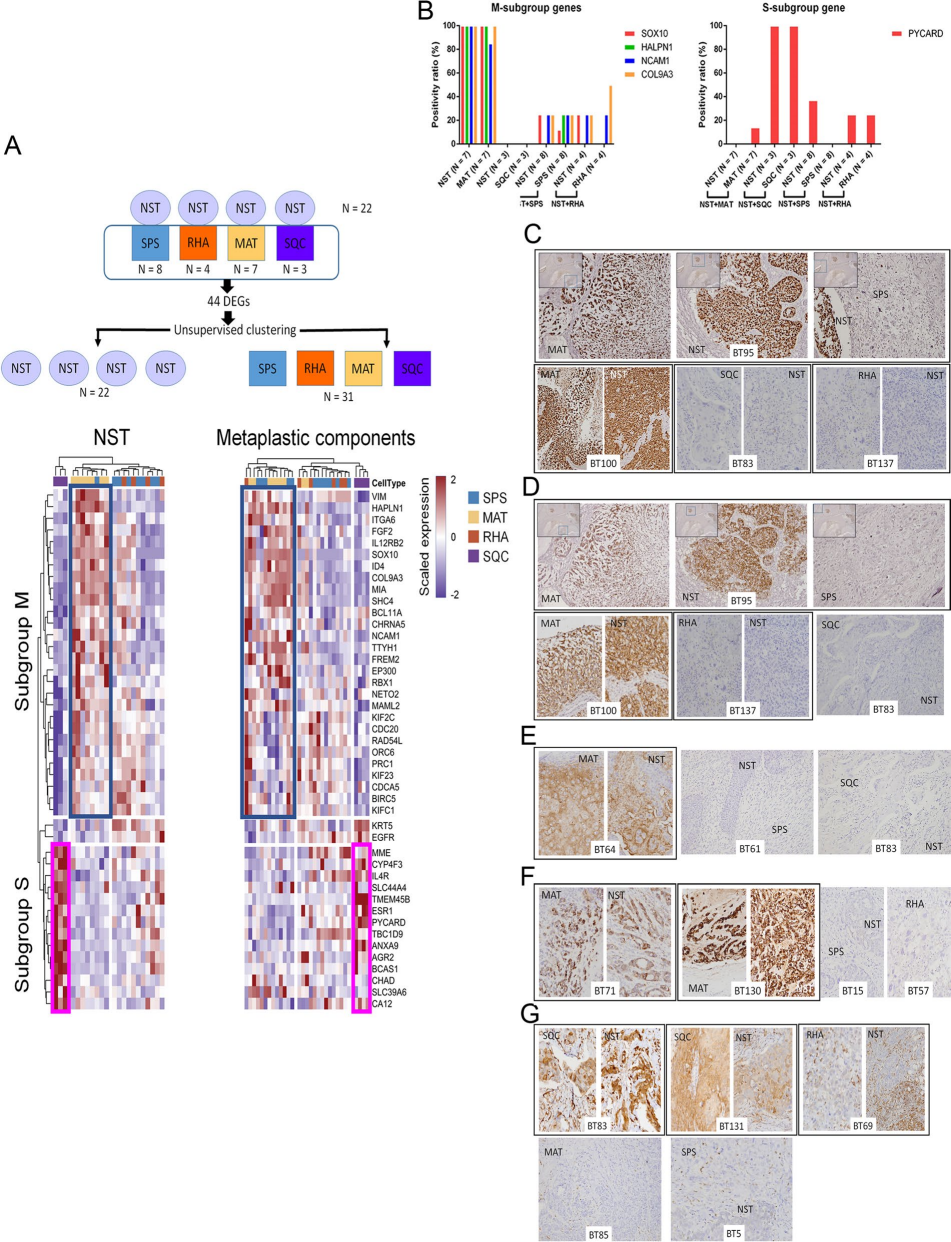
Ability of the intrinsic gene expression of NST to predict metaplastic components. **A** Unsupervised clustering of 22 NST components (left) and 31 metaplastic components (right) using 44 differentially expressed genes (ANOVA, *p* < 0.01). The clustering of NST components was according to NST samples categorized by corresponding metaplastic components. Genes majorly expressed in the MAT and SQC components are highlighted as subgroups M and S. DEGs, differentially expressed genes. B Bar plots of the immunohistochemical analysis of M-subgroup genes (*SOX10*, *HAPLN1*, *NCAM1*, and *COL9A3*) and S-subgroup gene (*PYCARD*) in the 22 MpBC cases with only one type or predominantly one type of paired metaplastic component. Representative pictures of immunohistochemical staining for C SOX10, D NCAM1, E HAPLN1, F COL9A3, and G PYCARD in the 27 MpBC cases. The NST and paired metaplastic components are marked with a black circle. The inset displays a low-power view. Positive PYCARD staining in some infiltrating mononuclear cells in the stroma

We next investigated whether metaplastic types were determined by the intrinsic gene expression of their paired NST components. We restricted our analysis to the 22 NST components with only one type (*n* = 19) or predominantly one type (> 95%; *n* = 3) of a paired metaplastic component. As displayed in Fig. 6A, 44 differentially expressed genes were identified among the metaplastic types (ANOVA, *p* < 0.05). Organized according to these 44 genes, the 22 NST components were separated into three clusters dominated by two sets of genes, namely subgroups S and subgroup M (Additional file 9: Table S6). The clusters were highly correlated with types of associated paired metaplastic components as follows: MAT (M-high/S-low), SQC (M-low/S-high), and SPS/RHA. We further clustered the 31 metaplastic components on the basis of the same 44 genes and identified three clusters with reference to the genes in subgroups S and M. The analysis achieved an accuracy of 74.2% (23/31), although one RHA component and 5 SPS components were misclassified into the MAT cluster and two MAT components were misclassified into the SPS/RHA cluster. The differentially expressed genes among the 31 metaplastic components were employed in separating 31 metaplastic components modestly correlated with metaplastic type. However, under these gene sets, the 22 NST components could not be clustered with their corresponding paired metaplastic types (Additional file 1: Fig. S1). The 27 MpBC samples were subjected to immunohistochemical analysis for M-subgroup (SOX10, NCAM1, HAPLN1, and COL9A3) and S-subgroup (PYCARD) proteins (Fig. 6B–G and Additional file 10: Table S7). Of the 27 MpBC cases, 22 had only one type or predominantly one type of paired metaplastic component. In nearly all MpBC cases with the MAT component, the coexpression of all four M-subgroup genes was observed in the MAT and NST components. In MpBC cases with the SQC component, the coexpression of the S-subgroup gene was observed in the SQC and NST components. However, in MpBC cases with SPS or RHA metaplasia, both M-subgroup and S-subgroup genes were generally not or less frequently expressed in the NST and metaplastic SPS and RHA components. A similar trend was observed in MpBC cases with multiple metaplastic components. The immunohistochemistry results were consistent with those of intrinsic gene clustering. To validate the significance of the intrinsic gene sets, 28 frozen samples from GSE57544 [12] were scored using gene set variation analysis (GSVA) against the M-subgroup and S-subgroup genes. Although no paired NST components were available for analysis, a high MAT and SQC signature score was observed in the chondroid and squamous components, respectively (Additional file 2: Fig. S2), thereby supporting our finding. Together, the observations presented suggest that the intrinsic gene expression of NST determines the metaplastic type.

### Correlation of EMT activity and stem cell traits in NST with lymph node metastasis

**Fig. 7:**
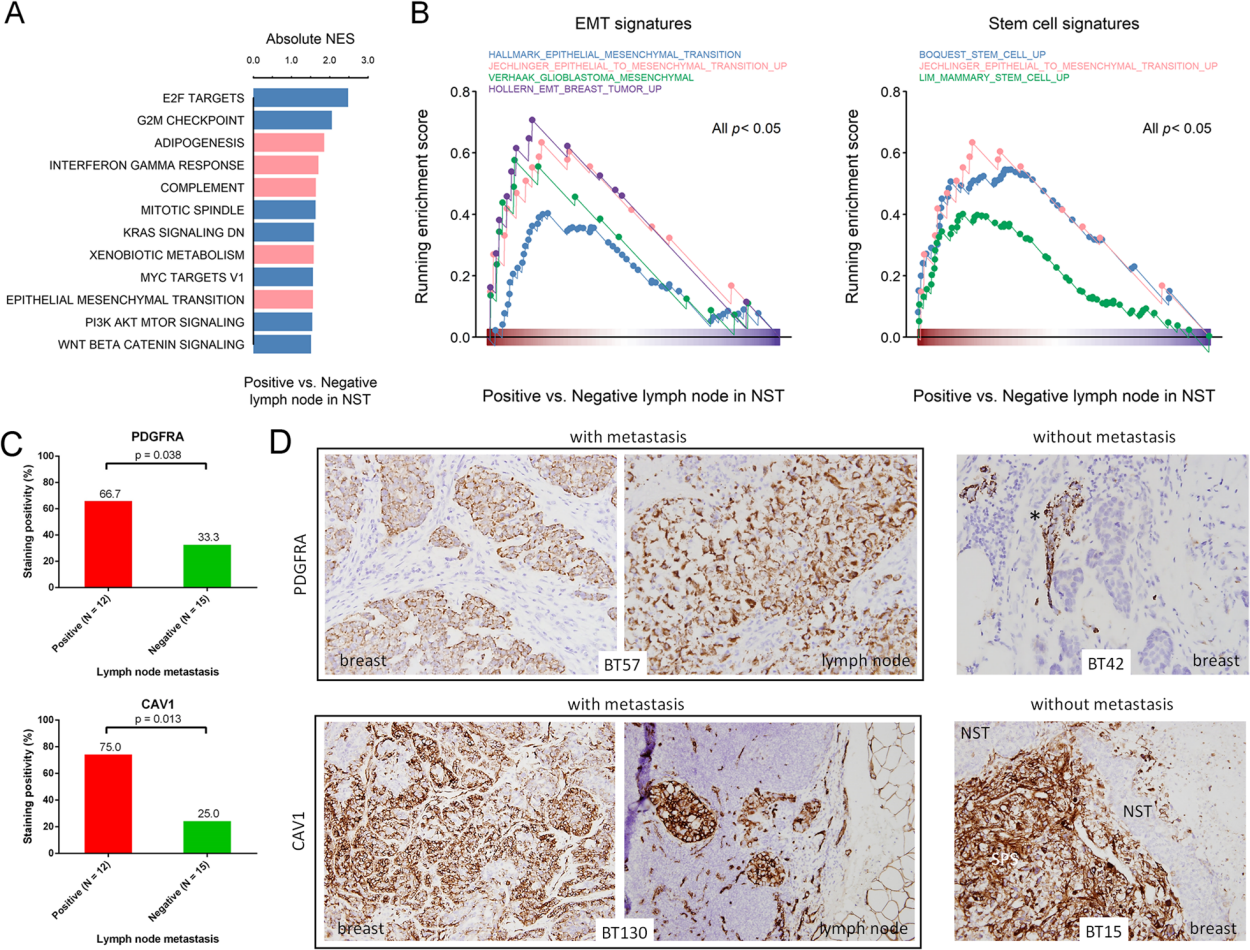
Functions associated with nodal metastasis. **A** Bar graph of significant MSigDB hallmark gene sets associated with NST with nodal metastasis, obtained through GSEA (*p* < 0.05). Bar colors indicate positive (pink) or negative (blue) values of normalized enrichment scores. **B** GSEA of gene sets related to the EMT and stem cells in NST specimens with and without nodal metastasis. **C** Bar plots representing the immunohistochemical verification of PDGFRA and CAV1 expression in the 27 MpBC cases with or without lymph node metastasis (chi-square test). **D** Representative pictures of immunohistochemical staining for PDGFRA and CAV1. Positive staining in ≥ 5% of tumor cells is considered positive. PDGFRA staining in the myoepithelial (star) cells surrounding the nonneoplastic mammary ducts, which serves as an internal positive staining control. CAV1 staining is also observed in vessels. In cases BT15, CAV1 staining is completely negative in the NST component, in contrast to the intense staining in the SPS component

Because the carcinomatous component was the predominant one present in metastatic axillary lymph nodes in the MpBC cases, we investigated whether any specific GEP in the NST was linked to nodal metastasis. The comparisons of GSEA of hallmark gene sets of NST components between cases with and without nodal metastasis revealed that cell cycle-related and cell proliferation-related pathways were negatively enriched in tumors with nodal metastasis (Fig. 7A). However, genes linked to the EMT and stem cells tended to be upregulated in tumors with nodal metastasis (Fig. 7A, B and Additional file 11: Table S8). Among these genes, *CAV1*, which is functionally associated with stem cell upregulation, and *PDGFRA*, which is functionally associated with EMT and stem cell upregulation, were selected for immunohistochemical verification in the 27 MpBC tissues. PDGFRA and CAV1 expression were both significantly more frequent in the NST components of MpBC cases with lymph node metastasis than in the NST components of those without metastasis (Fig. 7C, D), supporting the GSEA results. Because most NST components of MpBC are negative for ER, PR, and HER2, we validated the biological significance of the metastasis-associated genes by using data on triple-negative breast cancers from the Cancer Genome Atlas (TCGA). The results indicated a worse progression-free and overall survival in cases with high expression of the metastasis-associated genes (Additional file 3: Fig. S3). This finding supports the biological significance of the metastasis-associated genes.

## Discussion

Herein, we employed a hybridization-based method by using the NanoString BC360 Panel to examine the transcriptomic features of 59 microdissected samples of NST components and paired metaplastic components on specimens obtained from 27 patients with MpBC. We observed that distinct transcriptomic alterations may underlie metaplasia into histologically distinct metaplastic components. The heterogeneity of the intercase gene expression in the NST components, as highlighted by the PCA plots and the hierarchical clustering heat map, substantiates the need for a comparison of paired samples when exploring transcriptomic features underlying distinct metaplastic processes. The consistency rate of 94.9% (56/59) between the classification of molecular intrinsic subtypes of PAM50 and the immunohistochemistry/FISH results of the 59 NST and metaplastic components supports the validity of the analysis (Table 1).

Compared with the paired NST components, the SPS components demonstrated the upregulation of genes related to stem cells, and the EMT, and displayed enrichment in claudin-low, and TGF-β signatures. The claudin-low subtype was characterized by the high expression of EMT-related and stem cell-like genes and the low expression of cell–cell adhesion genes [22–24]. Furthermore, TGF-β signaling was found to play a critical role in the EMT [25]. A comparison of the GEPs of the SPS components and paired NST components confirmed the contributions of the EMT and claudin-low signatures to spindle cell metaplasia in MpBCs [11–13]. In addition, we observed the enrichment of macrophage signatures and the immune inhibitory genes *PD-L2* and *B3-H3* in the SPS components as well as the downregulation of the immune-related gene *TIGIT*. Immune microenvironments were reported as being distinct within different histological components. For example, the number of tumor-infiltrating lymphocytes (TILs) in sarcomatous components is generally lower than that in paired carcinomatous components [26]. Whether the differentially expressed signatures and genes herein explain the difference in the microenvironments between the carcinomatous and sarcomatous components warrants further study. Notably, the SPS components exhibited the downregulation of various genes involved in nucleosome organization and the cell cycle. The perturbation of chromatin remodeling complexes in malignant progression has been documented [27, 28]. Our findings suggest that such perturbations are involved in spindle metaplasia and are coordinating with EMT-related and stem cell-upregulated genes to contribute to an aggressive tumor phenotype.

RHA morphology, which features round to polygonal cells with eccentric nuclei and abundant eosinophilic cytoplasm, is occasionally observed as a metaplastic component in MpBCs [2]. Compared with those of other types of metaplasia, the gene expression of RHA metaplasia is less well understood. Herein, the enriched gene functions and signatures of the RHA components were somewhat similar to those of the SPS components. Specifically, they exhibited the upregulation of genes functionally related to cell adhesion, cell development, stem cells, and the EMT as well as the upregulation of claudin-low and macrophage signatures and the downregulation of differentiation signatures. Notably, despite some overlap between the RHA and SPS components in the enriched functions and signatures, the specific differentially expressed genes differed between these two types of metaplastic components (Fig. 4C). In the RHA components, we noted the RHA-specific upregulation of genes associated with VEGF signaling and the downregulation of genes enriched in cell adhesion. Moreover, a lack of alteration in genes related to nucleosome organization and the cell cycle, which were downregulated in the SPS components, was detected. These findings suggest that the GEPs of the RHA and SPS components are distinct yet overlapping. Our finding of the enrichment of EMT and claudin-low signatures in cases of MpBC with spindle and RHA components, but not in those featuring other metaplasia, may have clinical implications. A prior study using multiple independent datasets of patients who received neoadjuvant chemotherapy demonstrated that the pathological complete response rate was lower in claudin-low subtype than in basal-like subtypes [22]. Furthermore, MpBCs with spindle metaplasia in particular have an aggressive behavior [5]. The shared transcriptomic features of RHA and spindle metaplasia suggest that MpBC with RHA metaplasia has relative chemoresistance and a poor prognosis.

Several MAT-specific upregulated genes were related to hypoxia. Furthermore, the expression of hypoxia-responsive genes was relatively high in the MAT components compared to that in the NST components. Hypoxia is essential for extracellular matrix synthesis in cartilage, a highly hypoxic tissue [29]. Consistent with this evidence, all nine MAT components had chondroid metaplasia. Several MAT-upregulated genes were related to apoptosis, which was shown to be linked to hypoxia [30]. By contrast, genes related to the cell cycle were downregulated in the MAT components. For example, *SPRY1* facilitates cell cycle progression and suppresses cell apoptosis [31]. Moreover, hypoxia has been demonstrated to induce cell cycle arrest. Taken together, the evidence indicates that hypoxia contributes to matrix metaplasia in MpBCs. Compared with those in the paired NST components, the immune-related MHC2 signature, which measures the levels of human leukocyte antigen involved in the presentation of MHC class II antigens, was significantly downregulated in the MAT components. Also significantly downregulated was *TIGIT*, which encodes an immune receptor present in some T cells and natural killer cells. These observations echo those of a recent proteomic study reporting that inflammatory responses in MAT components are less active than those in spindle and squamous MpBCs [13]. In line with this finding, the proportion of high- or intermediate-level TILs was lower in MAT components than in paired NST components [26]. Taken together, the evidence indicates that the microenvironment in MAT components is relatively immune cold.

Herein, compared with genes linked to other types of metaplasia, fewer SQC differentially expressed genes (four upregulated, three downregulated) were observed. This may be partially explained by the small number of SQC components (*n* = 4). Alternatively, despite the histomorphological differences between SQC and NST components, differences in the gene expression of carcinomatous (SQC vs. NST) components might be smaller than those between sarcomatous and carcinomatous components. This is supported by the fact that GEP differences between NST components and paired SPS, RHA, or MAT components were greater than those between NST components and paired SQC components, as revealed in the PCA (Fig. 3D). Nevertheless, the SQC components demonstrated the upregulation of genes related to apoptosis, immune responses, and cell adhesion (Fig. 4C). The finding that SQC-specific upregulation genes were functionally associated with cell adhesion is consistent with the prior proteomic study demonstrating the upregulation of cell adhesion markers in squamous MpBCs [13]. The SQC components displayed upregulation of the TGF-β signature (Fig. 5A, D), which modulates processes such as immune regulation and microenvironment modification in cancers. These findings suggest that the upregulation of apoptosis, immune responses, and cell adhesion, along with microenvironment modification, are potential GEPs underlying squamous metaplasia in MpBCs.

Whether the intrinsic GEP of NST determines the type of metaplasia occurring in MpBCs remains unclear. In the present study, the differentially expressed genes among the metaplastic components obtained from the 22 MpBC cases with only one or predominantly one type of metaplastic component could separate the paired 22 NST samples with correlation with their associated metaplastic types. Notably, these genes were employed in separating the 31 metaplastic components according to their respective metaplastic types, and the accuracy rate obtained was 74.2%. These results were consistent with those of immunohistochemical analysis, thereby strengthening the link between NST and paired metaplastic components and indicating that the intrinsic gene expression of NST may determine the metaplastic type.

We also evaluated PAM50 ROR scores derived from the BC360 Panel in the NST components and metaplastic components. The ROR scores varied with histological components, with the majority of cases demonstrating scores higher than those of the paired NST components in the SPS and RHA components. Moreover, in the majority of cases, the scores in the MAT and SQC components were lower than those in the paired NST components. These findings may have prognostic implications. Specifically, the ROR scores for patients with MpBC may vary with the histological components from which the tumor specimens were collected. These findings highlight the effects of histology-related heterogeneity on transcriptomic signatures and prognostic information in MpBCs. In addition, the enrichment of claudin-low signature in the SPS and RHA components in our study, along with the EMT-like transcriptomic profiles and the high prevalence of the claudin-low subtype in MpBC with spindle cell metaplasia demonstrated in previous studies [11–13], support the assumption that the enrichment of EMT or claudin-low signatures in MpBCs stems from the analyzed SPS or RHA components [11–13, 32–34].

Although MpBCs have been shown to reveal genetic heterogeneity that broadly correlates with histologic subtype [7], nearly identical landscapes of somatic mutation of paired invasive ductal carcinoma and metaplastic tumor component suggests epigenetic or noncoding changes may mediate the metaplastic phenotype of MpBCs [10]. Our finding of the distinct transcriptomic alterations underlying metaplasia into specific metaplastic components in MpBCs is in line with this notion. One limitation of our study is that only 770 genes relevant to the well-known critical biology of breast cancer were analyzed. Nevertheless, the expression of several essential signatures defined in the BC360 panel, including p53, proliferation, and homologous recombination repair signatures, did not significantly differ between the NST and metaplastic components. This indicates that, although certain transcriptomic alterations may correlate with metaplasia, some tumor-intrinsic key traits may persist in NST components and metaplastic components. In addition, the metaplasia-related transcriptomic alterations do not seem to involve TP53, PI3K, and MAPK pathways where genes of these pathways are frequently mutated in MBpCs [7]. This suggests that drivers of initiators of MpBC may not involve in the metaplastic process. Identification of potential mechanisms such as epigenetic or noncoding changes that result in these transcriptomic alterations will be critical for understanding the pathogenesis underlying the metaplastic processes of MBpCs.

The majority of MpBCs are triple-negative; however, they demonstrate axillary lymph node metastasis less frequently than conventional TNBC [35]. In addition, when metastatic foci in the lymph nodes are present in MpBCs, they tend to consist of carcinomatous rather than sarcomatous components [1, 36]. Similar findings were observed in uterine carcinosarcoma [37]. Consistent with this evidence, 10 cases of MpBC with mixed carcinomatous and sarcomatous components in the present study exhibited lymph node metastasis. Seven of these cases featured only carcinomatous deposits in the lymph nodes, whereas the remaining three cases featured both carcinomatous and sarcomatous components, with the carcinomatous components being predominant. Notably, none of the 10 cases exhibited only sarcomatous components in the metastatic lymph nodes. To elucidate the pathogenesis associated with nodal metastasis in the carcinomatous components, we conducted a GSEA of hallmark gene sets from MSigDB, observing that genes related to the EMT and stem cells tended to be upregulated in NST with nodal metastasis. Among these genes, PDGFRA and CAV1 expression were significantly more frequent in the NST components of the MpBC cases with lymph node metastasis than in the NST components of those without metastasis, thereby supporting the GSEA results. In line with findings on the role of EMT and the nature of stem cells in cancer dissemination, including lymph node metastasis, our finding indicates that EMT activity and stem cell traits in NST are correlated with lymph node metastasis in MpBCs [38–40]. Alternatively, the EMT signature, which was enriched in the SPS and RHA components, may be associated with the hematogenous (but not nodal) metastasis most often observed in these metaplastic components [35, 37]. This suggests that EMT activity can play roles in distinct dissemination patterns among different histologic components in MpBCs.

In summary, we presented distinct yet overlapping transcriptomic alterations underlying metaplasia into histologically distinct metaplastic components. Moreover, we provided evidence suggesting that the intrinsic signatures of NST may determine paired metaplastic types. The findings provide insight into the pathogenesis underlying the histologically distinct metaplasia observed in MpBCs.

## Supplementary Information

**Additional file 1**. **Fig. S1**: Unsupervised clustering of 31 metaplastic components (left) and 22 NST components (right) using the set of 126 differentially expressed genes among the 31 distinct metaplastic components (ANOVA, *p* < 0.01).

**Additional file 2**.** Fig. S2**: Validation of M-subgroup and S-subgroup gene sets in MpBC samples from GSE57544 (Weigelt B, Ng CK, Shen R et al. Mod Pathol 2015, 28(3), 340–351).

**Additional file 3**. **Fig. S3**: Differentially expressed genes between NST components with and without nodal metastasis and their impact on survival.

**Additional file 4**. **Table S1**: The treatment and outcome of the patients.

**Additional file 5**. **Table S2**: GEPs of the 59 samples of NST components and paired metaplastic components.

**Additional file 6**. **Table S3**: Differences in differentially expressed genes and gene sets between the NST components and paired metaplastic components (see also Fig. 4).

**Additional file 7**. **Table S4**: List of log2 fold changes of the BC360-defined signatures and genes of the NST versus paired metaplastic components.

**Additional file 8**. **Table S5**: Scores of BC360-defined signatures and genes in four cases of MpBC with multiple metaplastic components.

**Additional file 9**. **Table S6**. Gene lists of subgroups M and S.

**Additional file 10**. **Table S7**: Immunohistochemical staining for M-subgroup and S-subgroup proteins in the 27 MpBC samples.

**Additional file 11**. **Table S8**: Genes linked to nodal metastasis (p < 0.05) in the NST components and their functional relation to EMT and stem cells.

## Data Availability

The raw data are available from the corresponding author upon reasonable request.

## Abbreviations

ADM: Adrenomedullin
ANOVA: Analysis of variance
ARID1A: AT-rich interactive domain-containing protein 1A
BAX: Bcl-2-associated X protein
BBC3: Bcl-2-binding component 3
BCL2A1: Bcl-2-related protein A1
BDNF: Brain-derived neurotrophic factor
BNIP3: Bcl-2-interacting protein 3
CAV1: Coveolin-1
CDC7: Cell division cycle 7
COL2A1: Collagen type II alpha 1 chain
COL9A3: Collagen Type IX Alpha 3 Chain
EFNA3: Ephrin A3
ERBB2: Erb-B2 receptor tyrosine kinase 2
EMT: Epithelial–mesenchymal transition
EPAS1: Endothelial PAS domain protein 1
ER: Estrogen receptor
FBN1: Fibrillin-1
FFPE: Formalin-fixed, paraffin-embedded
FGFR3: Fibroblast growth factor receptor 3
FISH: Fluorescence in situ hybridization
GEP: Gene expression profile
GSEA: Gene set enrichment analysis
HAPLN1: Hyaluronan and proteoglycan link protein 1
HER2: Human epidermal growth factor receptor 2
HIST1H1C: Histone H1.2
HIST3H2BB: Histone H2B type 3-B
HMGA1: High-mobility group AT-hook 1
IL1RA: Interleukin 1 receptor antagonist
IL20RB: Interleukin 20 receptor subunit beta
INHBB: Inhibin subunit beta B
MAT: Chondroid matrix-producing
MDM2: Mouse double minute 2 homolog
MHC2: Major histocompatibility complex 2
MIS18A: MIS18 Kinetochore Protein A
MpBC: Metaplastic breast carcinoma
MSigDB: Molecular signatures database
NCAM1: Neural cell adhesion molecule 1
NOD2: Nucleotide binding oligomerization domain containing 2
NST: Invasive carcinoma of no special type
OGS: Osteoid matrix-producing
PCA: Principal component analysis
PDGFRA: Platelet-derived growth factor receptor alpha
PIK3R3: Phosphoinositide-3-Kinase Regulatory Subunit 3
PLCB1: 1-Phosphatidylinositol-4,5-bisphosphate phospholipase beta-1
PR: Progesterone receptor
PRKAA2: Protein kinase AMP-activated catalytic subunit alpha 2
PSMB7: Proteasome 20S subunit beta 7
PYCARD: PYD and CARD Domain Containing
RHA: Rhabdoid
ROR: Risk of recurrence
SLC2A1: Solute carrier family 2 member 1
SPRY1: Sprouty RTK signaling antagonist 1
SOX10: SRY-related HMG-box 10
SPS: Spindle carcinomatous
SQC: Squamous carcinomatous
TGF-β: Transforming growth factor-beta
TIL: Tumor-infiltrating lymphocyte
TIGIT: T cell immunoreceptor with Ig and immunoreceptor tyrosine-based inhibitory motif domains
TNBC: Triple-negative breast cancers
VEGF: Vascular endothelial growth factor
WEE1: WEE1 G2 checkpoint kinase
XRCC2: X-ray repair cross-complementing 2
