# Supplementary material for: Transcriptomic alterations underlying metaplasia into specific metaplastic components in metaplastic breast carcinoma
Source: Breast Cancer Res. 2023 Jan 27;25:11. doi: 10.1186/s13058-023-01608-5 (PMC9883935; doi:10.1186/s13058-023-01608-5)
Supplement: Supplementary file 1 — Additional file 1. Fig. S1: Unsupervised clustering of 31 metaplastic components (left) and 22 NST components (right) using the set of 126 differentially expressed genes among the 31 distinct metaplastic components (ANOVA, p < 0.01). [file 13058_2023_1608_MOESM1_ESM.docx]

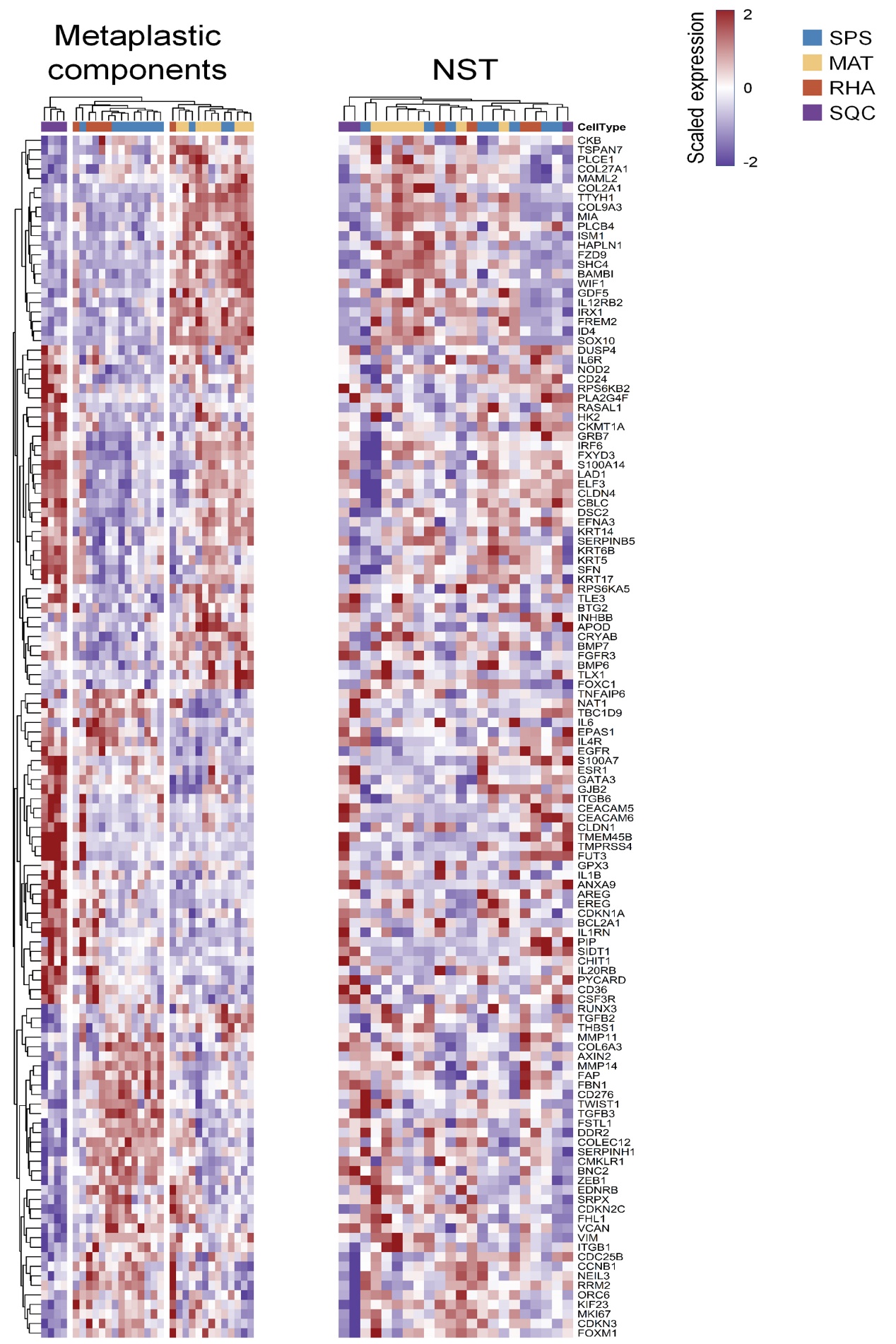


**Supplementary Fig. S1** Unsupervised clustering of 31 metaplastic components (left) and 22 NST components (right) using the set of 126 differentially expressed genes among the 31 distinct metaplastic components (ANOVA, *p <* 0.01).
