# Supplementary material for: Transcriptomic alterations underlying metaplasia into specific metaplastic components in metaplastic breast carcinoma
Source: Breast Cancer Res. 2023 Jan 27;25:11. doi: 10.1186/s13058-023-01608-5 (PMC9883935; doi:10.1186/s13058-023-01608-5)
Supplement: Supplementary file 2 — Additional file 2. Fig. S2: Validation of M-subgroup and S-subgroup gene sets in MpBC samples from GSE57544 (Weigelt B, Ng CK, Shen R et al. Mod Pathol 2015, 28(3), 340–351). [file 13058_2023_1608_MOESM2_ESM.docx]

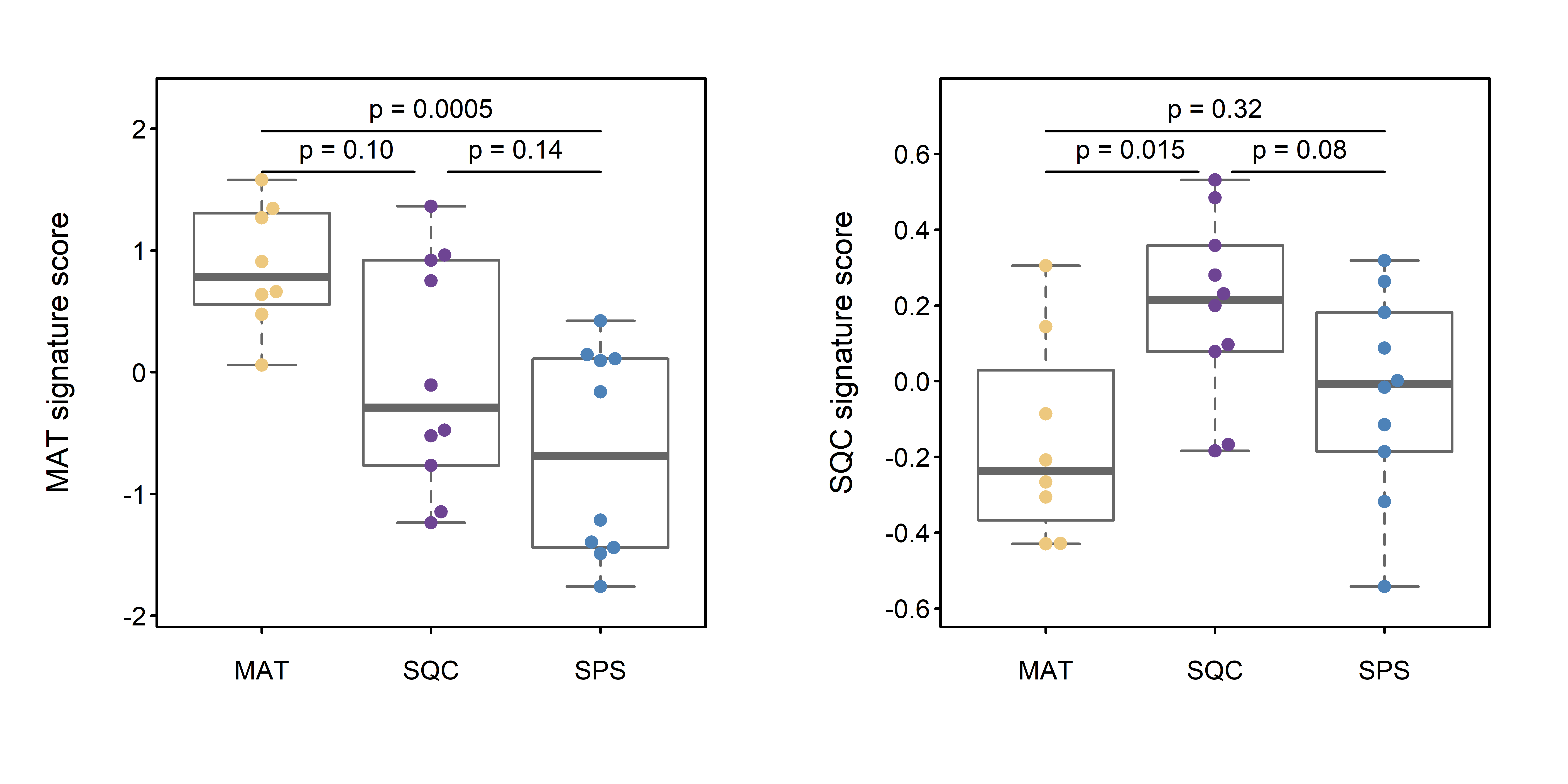


Chondroid Squamous Spindle

Chondroid Squamous Spindle

**Supplementary Fig. S2** Validation of M-subgroup and S-subgroup gene sets in MpBC samples from GSE57544 (Weigelt B, Ng CK, Shen R et al. Mod Pathol 2015, 28(3), 340-51). Twenty-eight frozen samples from GSE57544 were scored using gene set variation analysis (GSVA) against the M-subgroup and S-subgroup genes. The most abundant metaplastic component in each frozen sample was defined. p, Wilcoxon signed-rank test.
