# Supplementary material for: Transcriptomic alterations underlying metaplasia into specific metaplastic components in metaplastic breast carcinoma
Source: Breast Cancer Res. 2023 Jan 27;25:11. doi: 10.1186/s13058-023-01608-5 (PMC9883935; doi:10.1186/s13058-023-01608-5)
Supplement: Supplementary file 3 — Additional file 3. Fig. S3: Differentially expressed genes between NST components with and without nodal metastasis and their impact on survival. [file 13058_2023_1608_MOESM3_ESM.docx]

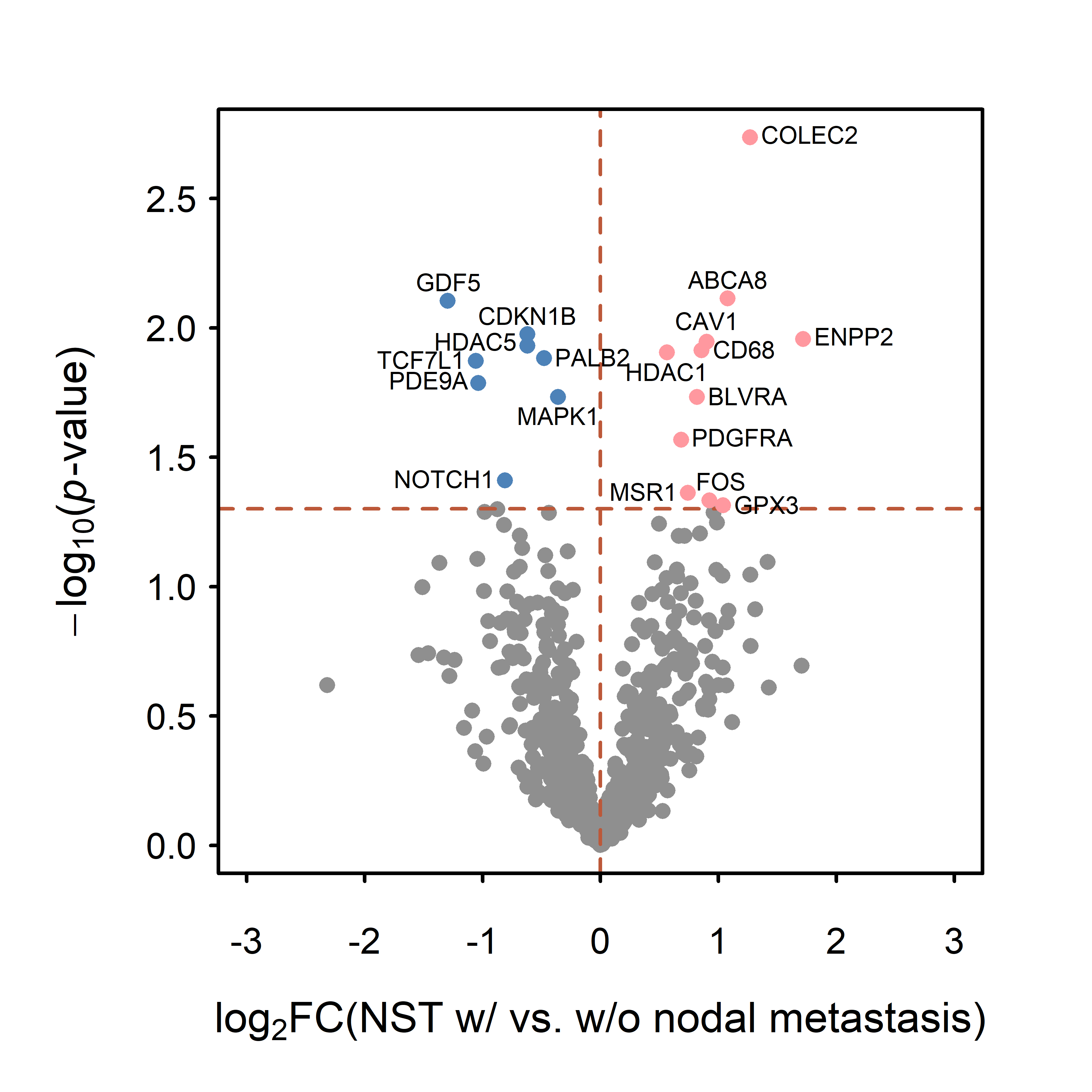

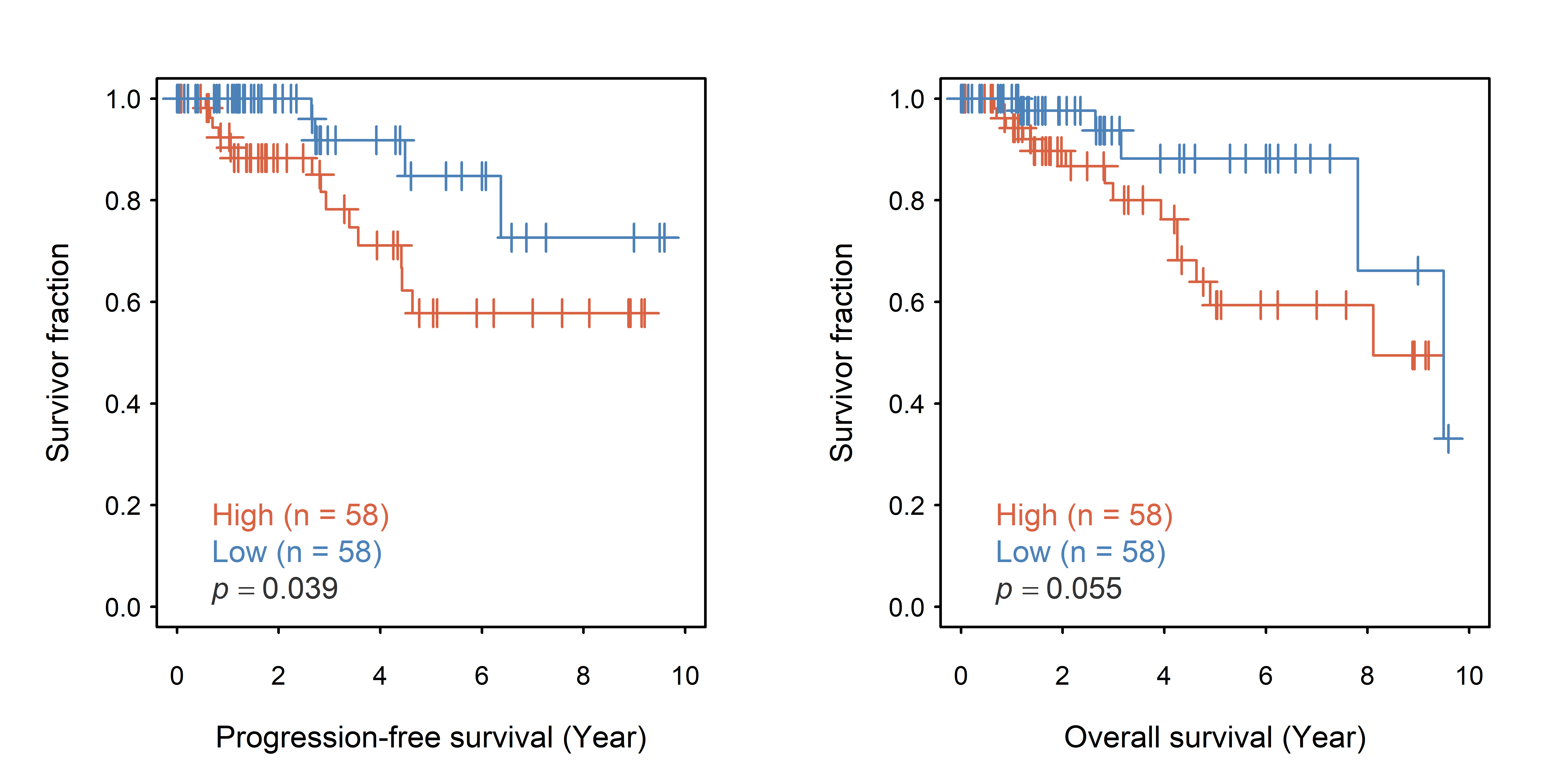


A

B

**Supplementary Fig. S3** Differentially expressed genes between NST components with and without nodal metastasis and their impact on survival. **A** Volcano plot representing the differentially expressed genes between NST components with and without nodal metastasis. Red and blue dots represent the significantly upregulated and downregulated genes, respectively. **B** Kaplan–Meier plots revealing the progression-free and overall survival between TCGA triple-negative breast cancers with high and low expression of metastasis-associated genes (red dots in A). Samples were scored using gene set variation analysis (GSVA) on the basis of the expression value of the metastasis-associated genes and stratified into high-expression and low-expression groups by the median value. p, log-rank test.
