# Supplementary material for: Transcriptomic alterations underlying metaplasia into specific metaplastic components in metaplastic breast carcinoma
Source: Breast Cancer Res. 2023 Jan 27;25:11. doi: 10.1186/s13058-023-01608-5 (PMC9883935; doi:10.1186/s13058-023-01608-5)
Supplement: Supplementary file 4 — Additional file 4. Table S1: The treatment and outcome of the patients. [file 13058_2023_1608_MOESM4_ESM.docx]

| **Supplementary Table S1.** The treatment and outcome of the patients | | | | | | | | | |
| --- | --- | --- | --- | --- | --- | --- | --- | --- | --- |
| **Sample No.** | **Date of diagnosis** | **Neoadjuvant chemotherapy (Y=1, N=0, Unknown=2)** | **Operation (Y=1, N=0, Unknown=2)** | **Adjuvant chemotherapy (Y=1, N=0, Unknown=2)** | **Adjuvant radiotherapy (Y=1, N=0, Unknown=2)** | **Recurrence (Y=1, N=0)** | **Relapse-free survival (m)** | **Alive till 2020/04/10 (Y=1, N=0, Unknown=2)** | **Overall survival (m)** |
| BT5 | 2003/7/4 | 0 | 1 | 0 | 0 | 1 | 7.27 | 0 | 19.63 |
| BT34 | 2007/11/13 | 0 | 1 | 1 | 0 | 1 | 8.00 | 0 | 10.40 |
| BT42 | 2008/5/17 | 0 | 1 | 1 | 0 | 0 | 121.30 | 1 | 121.30 |
| BT46 | 2008/6/23 | 0 | 1 | 1 | 0 | 0 | 141.97 | 1 | 141.97 |
| BT61 | 2010/5/15 | 0 | 1 | 1 | 1 | 1 | 41.97 | 0 | 80.20 |
| BT65 | 2010/10/1 | 0 | 1 | 0 | 1 | 0 | 111.50 | 1 | 111.50 |
| BT91 | 2015/4/13 | 1 | 1 | 1 | 0 | 1 | 6.20 | 0 | 28.97 |
| BT95 | 2016/1/28 | 0 | 1 | 1 | 1 | 0 | 48.03 | 1 | 48.03 |
| BT15 | 2003/7/23 | 1 | 1 | 0 | 1 | 1 | 18.73 | 0 | 26.27 |
| BT118 | 2019/1/16 | 0 | 1 | 1 | 0 | 0 | 26.30 | 1 | 26.30 |
| BT122 | 2013/12/1 | 1 | 1 | 1 | 2 | 1 | 8.43 | 0 | 9.47 |
| BT127 | 2017/7/22 | 0 | 1 | 1 | 1 | 1 | 19.57 | 0 | 37.40 |
| BT3 | 1998/12/31 | 0 | 1 | 1 | 0 | 1 | 5.13 | 0 | 5.97 |
| BT83 | 2012/7/30 | 1 | 1 | 0 | 1 | 1 | 20.40 | 0 | 28.17 |
| BT131 | 2018/3/9 | 0 | 1 | 1 | 0 | 0 | 305.03 | 1 | 35.03 |
| BT23 | 2005/7/5 | 0 | 1 | 1 | 0 | 0 | 178.23 | 1 | 178.23 |
| BT57 | 2009/12/9 | 0 | 1 | 0 | 1 | 1 | 14.70 | 0 | 63.37 |
| BT69 | 2011/10/25 | 0 | 1 | 0 | 1 | 1 | 27.83 | 0 | 74.03 |
| BT137 | 2018/8/9 | 1 | 1 | 0 | 0 | 1 | 13.30 | 1 | 20.57 |
| BT79 | 2011/12/16 | 0 | 1 | 1 | 1 | 0 | 100.13 | 0 | 100.13 |
| BT6 | 1998/11/30 | 0 | 1 | 1 | 0 | 0 | 260.23 | 1 | 260.23 |
| BT44 | 2008/6/11 | 0 | 1 | 1 | 0 | 0 | 144.50 | 1 | 144.50 |
| BT64 | 2010/11/15 | 0 | 1 | 1 | 1 | 0 | 38.10 | 2 | 38.10 |
| BT71 | 2012/7/4 | 0 | 1 | 1 | 0 | 0 | 91.17 | 1 | 91.17 |
| BT85 | 2013/11/14 | 0 | 1 | 1 | 0 | 0 | 75.07 | 1 | 75.07 |
| BT100 | 2017/6/23 | 0 | 1 | 1 | 1 | 1 | 17.90 | 0 | 19.00 |
| BT130 | 2017/5/31 | 1 | 1 | 0 | 1 | 0 | 16.10 | 0 | 16.10 |
