# Supplementary material for: Transcriptomic alterations underlying metaplasia into specific metaplastic components in metaplastic breast carcinoma
Source: Breast Cancer Res. 2023 Jan 27;25:11. doi: 10.1186/s13058-023-01608-5 (PMC9883935; doi:10.1186/s13058-023-01608-5)
Supplement: Supplementary file 7 — Additional file 7. Table S4: List of log2 fold changes of the BC360-defined signatures and genes of the NST versus paired metaplastic components. [file 13058_2023_1608_MOESM7_ESM.docx]

**Supplementary Table S4.** List of log2-fold changes of the BC360-defined signatures and genes of the NST versus paired metaplastic components

| **Paired NST vs SPS** | | | **Paired NST vs. RHA** | | | **Paired NST vs. MAT** | | | **Paired NST vs. SQC** | | |
| --- | --- | --- | --- | --- | --- | --- | --- | --- | --- | --- | --- |
| **Signature** | **log2FC** | **P**  **value** | **Signature** | **log2 FC** | **P**  **value** | **Signature** | **log2FC** | **P value** | **Signature** | **log2FC** | **P**  **value** |
| Claudin-Low | -3.55 | 0.00 | Claudin-Low | -2.31 | 0.01 | Hypoxia | -1.37 | 0.11 | Proliferation | -1.64 | 0.39 |
| Stroma | -3.03 | 0.00 | Macrophages | -1.43 | 0.04 | Claudin-Low | -0.44 | 0.34 | Apoptosis | -1.53 | 0.28 |
| TGF-β | -1.77 | 0.00 | Hypoxia | -1.43 | 0.20 | PD-L1 | -0.40 | 0.19 | BRCA | -1.33 | 0.14 |
| Macrophages | -1.04 | 0.01 | Proliferation | -1.29 | 0.31 | TGF-β | -0.27 | 0.42 | Claudin-Low | -1.24 | 0.15 |
| IFNγ | -0.77 | 0.31 | Apoptosis | -0.61 | 0.56 | Apoptosis | -0.24 | 0.78 | Hypoxia | -1.21 | 0.10 |
| Hypoxia | -0.77 | 0.16 | Cytotoxic cells | -0.58 | 0.49 | Mast cells | -0.15 | 0.65 | IDO1 | -1.19 | 0.29 |
| PD-L2 | -0.71 | 0.04 | TGF-β | -0.49 | 0.09 | Stroma | 0.02 | 0.98 | PD-L1 | -1.16 | 0.14 |
| Inflammatory.  chemokines | -0.71 | 0.29 | PD-L2 | -0.45 | 0.42 | PD-L2 | 0.02 | 0.87 | Cytotoxicity | -1.04 | 0.25 |
| B7.H3 | -0.71 | 0.01 | p53 | -0.43 | 0.36 | HRD | 0.04 | 0.79 | TGF-β | -0.97 | 0.03 |
| APM | -0.44 | 0.37 | IFNγ | -0.42 | 0.74 | Macrophages | 0.04 | 0.81 | Treg | -0.56 | 0.42 |
| MHC2 | -0.44 | 0.51 | MHC2 | -0.40 | 0.24 | BRCA | 0.06 | 0.80 | Cytotoxic cells | -0.53 | 0.54 |
| Proliferation | -0.37 | 0.10 | TIS | -0.39 | 0.59 | ERBB2 | 0.08 | 0.62 | p53 | -0.51 | 0.45 |
| Mast cells | -0.24 | 0.48 | PD-L1 | -0.38 | 0.63 | AR | 0.16 | 0.69 | TIS | -0.46 | 0.63 |
| PD-L1 | -0.19 | 0.54 | APM | -0.35 | 0.67 | Differentiation | 0.17 | 0.16 | TIGIT | -0.34 | 0.66 |
| TIS | -0.17 | 0.55 | CD8 T cells | -0.09 | 0.92 | PGR | 0.20 | 0.31 | HRD | -0.29 | 0.60 |
| p53 | -0.14 | 0.44 | HRD | -0.08 | 0.82 | Endothelial cells | 0.26 | 0.28 | Macrophages | -0.24 | 0.75 |
| HRD | 0.09 | 0.64 | IDO1 | -0.07 | 0.95 | Treg | 0.34 | 0.21 | APM | -0.06 | 0.95 |
| ER-signaling | 0.19 | 0.32 | TIGIT | -0.07 | 0.94 | p53 | 0.34 | 0.18 | PD-L2 | 0.10 | 0.93 |
| Endothelial.cells | 0.19 | 0.37 | Inflammatory.  chemokines | 0.03 | 0.97 | Inflammatory chemokines | 0.36 | 0.59 | CD8 T cells | 0.14 | 0.81 |
| PGR | 0.22 | 0.27 | Endothelial cells | 0.15 | 0.55 | B7.H3 | 0.39 | 0.14 | MHC2 | 0.14 | 0.94 |
| Cytotoxicity | 0.27 | 0.70 | Cytotoxicity | 0.18 | 0.71 | IDO1 | 0.39 | 0.23 | Endothelial cells | 0.17 | 0.70 |
| Cytotoxic cells | 0.29 | 0.42 | B7.H3 | 0.18 | 0.46 | Cytotoxic.cells | 0.41 | 0.13 | IFNγ | 0.38 | 0.83 |
| Treg | 0.49 | 0.07 | BRCA | 0.46 | 0.43 | ER-signaling | 0.44 | 0.04 | B7.H3 | 0.44 | 0.45 |
| CD8 T cells | 0.51 | 0.14 | ER-signaling | 0.53 | 0.22 | IFNγ | 0.51 | 0.51 | Mast cells | 0.52 | 0.57 |
| Apoptosis | 0.52 | 0.41 | Treg | 0.53 | 0.39 | TIS | 0.51 | 0.05 | ER-signaling | 0.86 | 0.33 |
| AR | 0.60 | 0.20 | Mast cells | 0.78 | 0.25 | Proliferation | 0.52 | 0.22 | Differentiation | 1.02 | 0.17 |
| BRCA | 0.60 | 0.08 | Stroma | 1.06 | 0.51 | Cytotoxicity | 0.55 | 0.31 | ERBB2 | 1.02 | 0.23 |
| TIGIT | 0.69 | 0.05 | Differentiation | 1.13 | 0.04 | ESR1 | 0.61 | 0.09 | Stroma | 1.10 | 0.44 |
| IDO1 | 0.78 | 0.12 | ESR1 | 1.51 | 0.27 | APM | 0.62 | 0.21 | Inflammatory chemokines | 1.47 | 0.30 |
| ESR1 | 1.21 | 0.01 | PGR | 1.67 | 0.04 | CD8 T cells | 0.64 | 0.14 | PGR | 1.54 | 0.43 |
| ERBB2 | 1.34 | 0.00 | AR | 1.94 | 0.17 | TIGIT | 0.64 | 0.01 | ESR1 | 1.71 | 0.41 |
| Differentiation | 1.38 | 0.00 | ERBB2 | 2.81 | 0.03 | FOXA1 | 0.71 | 0.33 | AR | 2.40 | 0.05 |
| FOXA1 | 1.58 | 0.07 | FOXA1 | 3.30 | 0.12 | MHC2 | 1.25 | 0.01 | FOXA1 | 2.78 | 0.30 |

log2FC, log2 fold change (NST / metaplastic component)

Abbreviation: APM, antigen processing machinery; IDO1, indoleamine 2,3 dioxygenase 1; ERBB, Erb-B2 receptor tyrosine kinase 2; HRD, homologous recombination repair status; IFNγ, interferon γ; MHC2, MHC class II antigen presentation; PGR, progesterone receptor; TGF-β, transforming growth factor-β; TIGIT, T cell immunoreceptor and Ig and ITIMS domains; TIS, tumor inflammation signature; Treg, regulatory T cell abundance
