# Supplementary material for: Transcriptomic alterations underlying metaplasia into specific metaplastic components in metaplastic breast carcinoma
Source: Breast Cancer Res. 2023 Jan 27;25:11. doi: 10.1186/s13058-023-01608-5 (PMC9883935; doi:10.1186/s13058-023-01608-5)
Supplement: Supplementary file 8 — Additional file 8. Table S5: Scores of BC360-defined signatures and genes in four cases of MpBC with multiple metaplastic components. [file 13058_2023_1608_MOESM8_ESM.docx]

**Supplementary Table S5.** Scores of BC360-defined signatures and genes in four cases of MpBC with multiple metaplastic components

| **Claudin-low signature** | **High** | **Middle** | **Low** |
| --- | --- | --- | --- |
| BT15 | SPS (10.1532) | RHA ((9.3493) | NST (6.1603) |
| BT95 | SPS (8.5800) | MAT (6.9403) | NST (5.7974) |
| BT118 | SPS (9.8989) | MAT (7.2785) | NST (6.6953) |
| BT127 | SPS (9.5688) | SQC (4.9433) | NST (3.5687) |
| **TGF-β gene** | **High** | **Middle** | **Low** |
| BT15 | SPS (2.2644) | RHA (1.8179) | NST (0.7370) |
| BT95 | SPS (1.8833) | MAT (0.8299) | NST (0.0139) |
| BT118 | SPS (3.0748) | NST (0.7533) | MAT (0.2457) |
| BT127 | SPS (3.7395) | SQC (0.5551) | NST (-0.5818) |
| **Macrophage signature** | **High** | **Middle** | **Low** |
| BT15 | SPS (-0.4084) | RHA (-1.9361) | NST (-2.5580) |
| BT95 | SPS (-1.3262) | MAT (-2.3252) | NST (-2.9450) |
| BT118 | SPS (-1.5632) | NST (-1.8228) | MAT (-2.5243) |
| BT127 | SQC (-1.6538)) | SPS (-1.7622) | NST (-1.9800) |
| **Differentiation signature** | **High** | **Middle** | **Low** |
| BT15 | NST (3.3363) | SPS (2.3185) | RHA (1.8973) |
| BT95 | NST (3.4530) | MAT (2.8828) | SPS (2.2678) |
| BT118 | MAT (2.7501) | NST (2.6851) | SPS (2.4828) |
| BT127 | NST (4.8157) | SQC (4.6666) | SPS (2.8502) |
| **Hypoxia signature** | **High** | **Middle** | **Low** |
| BT15 | RHA (7.0028) | SPS (5.1574) | NST (4.7778) |
| BT95 | MAT (9.8911) | NST (8.2150) | SPS (2.2678) |
| BT118 | MAT (9.5738) | NST (6.9560) | SPS (6.6684) |
| BT127 | SQC (8.3102) | SPS (7.6728) | NST (6.4216) |
