# Supplementary material for: Transcriptomic alterations underlying metaplasia into specific metaplastic components in metaplastic breast carcinoma
Source: Breast Cancer Res. 2023 Jan 27;25:11. doi: 10.1186/s13058-023-01608-5 (PMC9883935; doi:10.1186/s13058-023-01608-5)
Supplement: Supplementary file 9 — Additional file 9. Table S6. Gene lists of subgroups M and S. [file 13058_2023_1608_MOESM9_ESM.docx]

**Supplementary Table S6.** Gene lists of subgroups M and S

| **Subgroup M** | **Subgroup S** |
| --- | --- |
| *VIM* | *CA12* |
| *HAPLN1* | *SLC39A6* |
| *TIGA6* | *CHAD* |
| *FGF2* | *BCAS1* |
| *IL2RB2* | *AGR2* |
| *SOX10* | *ANXA9* |
| *ID4* | *TBC1D9* |
| *COL9A3* | *PYCARD* |
| *MIA* | *ESR1* |
| *SHC4* | *TMEM45B* |
| *BCL11A* | *SLC44A4* |
| *CHRNA5* | *IL4R* |
| *NCAM1* | *CYP4F3* |
| *TTYH1* | *MME* |
| *FREM2* |  |
| *EP300* |  |
| *RBX1* |  |
| *NETO2* |  |
| *MAML2* |  |
| *KIF2C* |  |
| *CDC20* |  |
| *RAD54L* |  |
| *ORC6* |  |
| *PRC1* |  |
| *KIF23* |  |
| *CDCA5* |  |
| *BIRC5* |  |
| *KIFC1* |  |
