# Supplementary material for: Transcriptomic alterations underlying metaplasia into specific metaplastic components in metaplastic breast carcinoma
Source: Breast Cancer Res. 2023 Jan 27;25:11. doi: 10.1186/s13058-023-01608-5 (PMC9883935; doi:10.1186/s13058-023-01608-5)
Supplement: Supplementary file 10 — Additional file 10. Table S7: Immunohistochemical staining for M-subgroup and S-subgroup proteins in the 27 MpBC samples. [file 13058_2023_1608_MOESM10_ESM.docx]

**Supplementary Table S7.** Immunohistochemical staining for M-subgroup and S-subgroup proteins in the 27 MpBC samples

|  |  |  |  | **M-subgroup genes** | |  |  | **S-subgroup gene** |
| --- | --- | --- | --- | --- | --- | --- | --- | --- |
|  |  |  | **SOX10** | **HAPLN1** | **NCAM1** | **COL9A3** |  | **PYCARD** |
|  |  |  | **Case number of positive staining* (%)** | | | |  |  |
| NST with only one or predominantly one metaplastic component | | | | | |  |  |  |
|  | NST with MAT (N = 7) | |  |  |  |  |  |  |
|  |  | NST (N = 7) | 7 (100) | 7 (100) | 7 (100) | 7 (100) |  | 0 (0) |
|  |  | MAT (N = 7) | 7 (100) | 7 (100) | 6 (85.7) | 7 (100) |  | 1 (14.3) |
|  | NST with SQC (N = 3) | |  |  |  |  |  |  |
|  |  | NST (N = 3) | 0 (0) | 0 (0) | 0 (0) | 0 (0) |  | 3 (100) |
|  |  | SQC (N = 3) | 0 (0) | 0 (0) | 0 (0) | 0 (0) |  | 3 (100) |
|  | NST with SPS (N = 8) | |  |  |  |  |  |  |
|  |  | NST (N = 8) | 2 (25.0) | 0 (0) | 2 (25.0) | 2 (25.0) |  | 3 (37.5) |
|  |  | SPS (N = 8) | 1 (12.5) | 2 (25.0) | 2 (25.0) | 2 (25.0) |  | 0 (0) |
|  | NST with RHA (N = 4) | |  |  |  |  |  |  |
|  |  | NST (N = 4) | 1 (25.0) | 0 (0) | 1 (25.0) | 1 (25.0) |  | 1 (25.0) |
|  |  | RHA (N = 4) | 0 (0) | 0 (0) | 1 (25.0) | 2 (50.0) |  | 1 (25.0) |
| NST with multiple metaplastic components | | | |  |  |  |  |  |
|  | NST with MAT and SPS (N = 1) | |  |  |  |  |  |  |
|  |  | NST | + | + | + | + |  | - |
|  |  | MAT | + | + | + | + |  | - |
|  |  | SPS | - | - | - | + |  | - |
|  | NST with MAT, SPS and RHA (N = 1) | | |  |  |  |  |  |
|  |  | NST | + | - | - | - |  | - |
|  |  | MAT | - | - | - | - |  | - |
|  |  | SPS | - | - | - | - |  | - |
|  |  | RHA | - | - | + | - |  | - |
|  | NST with MAT, OGS and SPS (N = 1) | | |  |  |  |  |  |
|  |  | NST | + | + | + | + |  | - |
|  |  | MAT | + | + | + | + |  | - |
|  |  | OGS | + | - | + | + |  | - |
|  |  | SPS | - | - | - | + |  | - |
|  | NST with SPS and SQC (N = 1) | |  |  |  |  |  |  |
|  |  | NST | - | - | - | - |  | + |
|  |  | SPS | - | - | - | - |  | - |
|  |  | SQC | - | - | - | - |  | + |
|  | NST with RHA and SQC (N = 1) | |  |  |  |  |  |  |
|  |  | NST | - | - | - | - |  | + |
|  |  | RHA | - | - | - | + |  | - |
|  |  | SQC | - | - | - | - |  | + |

*Staining in ≧ 5% of tumor cells is considered positive for these five genes.
