# Supplementary material for: Transcriptomic alterations underlying metaplasia into specific metaplastic components in metaplastic breast carcinoma
Source: Breast Cancer Res. 2023 Jan 27;25:11. doi: 10.1186/s13058-023-01608-5 (PMC9883935; doi:10.1186/s13058-023-01608-5)
Supplement: Supplementary file 11 — Additional file 11. Table S8: Genes linked to nodal metastasis (p < 0.05) in the NST components and their functional relation to EMT and stem cells. [file 13058_2023_1608_MOESM11_ESM.docx]

**Supplementary Table S8.** Genes linked to nodal metastasis (p < 0.05) in the NST components and their functional relation to EMT and stem cells.

| **GeneName** | **log2FC*** | **p-value** | **HALLMARK_EPITHELIAL_MESENCHYMAL_TRANSITION** | **JECHLINGER_EPITHELIAL_TO_MESENCHYMAL_TRANSITION_UP** | **VERHAAK_GLIOBLASTOMA_MESENCHYMAL** | **HOLLERN_EMT_BREAST_TUMOR_UP** | **BOQUEST_STEM_CELL_UP** | **LIM_MAMMARY_STEM_CELL_UP** |
| --- | --- | --- | --- | --- | --- | --- | --- | --- |
| COLEC12 | 1.27 | 0.0018 | No | No | No | No | Yes | No |
| ABCA8 | 1.08 | 0.0077 | No | No | No | No | Yes | No |
| GDF5 | -1.29 | 0.0079 | No | No | No | No | No | No |
| CDKN1B | -0.62 | 0.0106 | No | No | No | No | No | No |
| ENPP2 | 1.72 | 0.011 | No | No | No | No | No | Yes |
| CAV1 | 0.90 | 0.0113 | No | No | No | No | No | Yes |
| HDAC5 | -0.62 | 0.0117 | No | No | No | No | No | No |
| CD68 | 0.86 | 0.0122 | No | No | No | No | No | No |
| HDAC1 | 0.56 | 0.0124 | No | No | No | No | No | No |
| PALB2 | -0.48 | 0.0131 | No | No | No | No | No | No |
| TCF7L1 | -1.06 | 0.0134 | No | No | No | No | No | Yes |
| PDE9A | -1.03 | 0.0163 | No | No | No | No | No | No |
| BLVRA | 0.82 | 0.0185 | No | No | No | No | No | No |
| MAPK1 | -0.36 | 0.0185 | No | No | No | No | No | No |
| PDGFRA | 0.68 | 0.0271 | No | Yes | No | Yes | Yes | No |
| NOTCH1 | -0.81 | 0.0389 | No | No | No | No | No | No |
| MSR1 | 0.74 | 0.0435 | No | No | Yes | No | No | No |
| FOS | 0.92 | 0.0465 | No | No | No | No | No | No |
| GPX3 | 1.04 | 0.0485 | No | No | No | No | No | No |

Log2FC*, log2 fold change (nodal metastasis / nodal non-metastasis)
